# Involvement of people who use alcohol and other drug services in the development of patient‐reported measures of experience: A scoping review

**DOI:** 10.1111/hex.13829

**Published:** 2023-07-29

**Authors:** Anke E. van der Sterren, Sally Nathan, Patrick Rawstorne, Elisabeth Yarbakhsh, Chris Gough, Devin Bowles

**Affiliations:** ^1^ Alcohol Tobacco and Other Drug Association ACT (ATODA) Canberra Australian Capital Territory Australia; ^2^ School of Population Health UNSW Sydney Sydney New South Wales Australia; ^3^ Canberra Alliance for Harm Minimisation and Advocacy (CAHMA) Canberra Australian Capital Territory Australia

**Keywords:** measure development, patient experience, patient satisfaction, person‐centred care, service user involvement

## Abstract

**Introduction:**

Patient‐reported measures that assess satisfaction and experience are increasingly utilised in healthcare sectors, including the alcohol and other drug (AOD) sector. This scoping review identifies how and to what extent people accessing AOD services have been involved in the development of satisfaction and experience measures to date.

**Methods:**

PubMed, EMBASE, CINAHL, Scopus, ProQuest, Google and Google Scholar were searched. Included papers described the development and/or implementation of a multiple‐item measure of patient‐reported experience or satisfaction specifically for people accessing AOD treatment and/or harm reduction programmes. If there was more than one paper, key papers were chosen that described each measure. The method of development, including service user involvement, was assessed against a framework generated for this review. Two reviewers were involved at each stage.

**Results:**

Thirty measures—23 satisfaction and 7 experience—were identified. Sixteen measures reported some level of involvement by people accessing AOD services in their development, although, for most measures, at a relatively low level. This involvement increased over the time span of the review becoming more frequent in later years. Only four measures were developed for use in harm reduction‐specific settings, and fewer than half reported undertaking analysis of underlying scale structure and constructs.

**Conclusion:**

Several gaps could be addressed to enhance the measurement of patient‐centred care in the AOD sector, including: developing experience measures for use in harm reduction settings and across various AOD settings in a service system; improved reporting of psychometric properties of these measures and increasing commitment to the meaningful involvement of AOD service users in measure development.

**Patient or Public Contribution:**

This scoping review is part of a broader codesign project that involves a partnership between the peak organisation for AOD services and the peer‐based AOD consumer organisation in the Australian Capital Territory, Australia. These organisations are working closely together to engage with AOD service users, service providers and policy makers in this codesign project. As such, the Executive Director of the peer‐based AOD consumer organisation is involved as a co‐author of this scoping review.

## 1 INTRODUCTION

Extensive work has occurred across diverse healthcare contexts internationally to embed ‘person‐centred’ healthcare—or a variation of this term—as a key dimension of high‐quality health service delivery.^1^, ^2^, ^3^ Patients' perspectives, and reliable tools to assess these, are therefore increasingly important to health services and health systems.^2^ Anonymous and standardised patient‐reported measures (PRMs) that enable self‐reporting of satisfaction and health experiences are increasing in prominence across the health sector,^4^, ^5^ and can assist in benchmarking to drive service quality improvements, and person‐centred care.^6^ The utility of PRMs is determined by their appropriateness to the service context type, and their inclusion of domains of experience relevant to the people accessing those services.^6^

PRMs of health service experience have, in the past, largely focused on examining ‘satisfaction’, where satisfaction is a measure of the reaction to the services received based on a subjective assessment against patient *expectations* of health care.^7^ While satisfaction is one element of experience, it is not the only one.^8^ Surveys that measure only satisfaction cannot identify problems with specific processes of health care, with findings not easily linked to planning processes and responses.^9^

More recently, PRMs have focused on examining ‘experiences’ or ‘perceptions of care’ more broadly, obtaining service user views on aspects of their health treatment and support, including their views on the accessibility and physical environment of services, and aspects of service user–clinician interactions.^10^ The focus is on describing what has happened during a patient's health care, rather than the more subjective evaluation of how satisfied they were with the care after they received it.^11^ The measurement of broader facets of experience, not just satisfaction, can enable a more comprehensive assessment of person‐centred health care.^8^, ^12^

### 1.1 Measuring patient‐reported satisfaction and experience in alcohol and other drug (AOD) services

Although not as advanced as in some other healthcare sectors, the AOD sector has been giving greater importance to person‐centred care.^13^ AOD service users are increasingly provided with active support in decision‐making around their treatment and given a voice in the treatment experience.^14^ Similarly, service users have improved opportunities to participate in the development of approaches to the measurement of their perceptions of healthcare quality.^15^ The utility of AOD service experience measures to provide valid and reliable assessments is not only determined through the quality of the survey design process but also through the inclusion of concepts relevant to the contexts and experiences of AOD service users.^6^ This means designing measures that include more than just ‘satisfaction’ and instead reflect broader dimensions of AOD service experiences that are meaningful to people who use AODs.

### 1.2 Measuring more than ‘satisfaction’

Although satisfaction measures have dominated the assessment of health services' quality in the AOD sector,^8^, ^9^ these measures may not adequately or accurately represent the experiences of people accessing AOD services. Satisfaction scores are typically high and may not reflect actual experiences, including experiences of unmet AOD treatment needs.^9^, ^16^ Some of the reasons for this mismatch are that service users may have low expectations of service provision or feel undeserving of a quality treatment experience. They may rate the service acceptable, even if the service experience would be objectively rated as poor. Low expectations of service provision are likely to be common in a group that experiences stigma and marginalisation associated with drug use and participation in AOD treatment.^16^ High satisfaction scores can also be indicative of the service user's dependent position in relation to service providers, in particular, the fear of losing access to treatment that is ‘vastly preferable to their lives without treatment’.^16^ ^,p.676^ Concerns about potential impacts on current or future treatment may be further exacerbated where service users perceive a lack of anonymity in their responses. Social etiquette may also affect satisfaction responses when service users feel they need to ‘maintain constructive social relationships with those caring for them’,^17^ ^,p.11^ or to show appreciation for the efforts of staff doing the best they can with limited resources.^9^ Conversely, a service may achieve positive outcomes, but service users may report low satisfaction because of the way they feel they have been treated.^18^ Survey measures that include concepts beyond ‘satisfaction’ will reflect a more complete experience of AOD treatment, which can then be used to improve care.

### 1.3 Measures that are relevant to the context of AOD treatment and harm reduction

Many measures of satisfaction or experience used in the AOD sector have been adapted from general health care or from the mental health sector, and many of these are not validated for use in AOD settings.^9^ While surveys such as the Client Satisfaction Questionnaire‐8^19^ and the Mental Health Statistics Improvement Program^20^ have been validated for use in AOD services, these were originally designed for use in general health and human services, and mental health settings, respectively. Such surveys, when adapted to the AOD context, are either used as is, with no changes or have minor wording changes to reflect the context (e.g., changing ‘mental health practitioner’ to ‘alcohol and other drug practitioner’). This translation is, however, not necessarily appropriate. Although ‘substance use disorders’ are classified by the medical profession as mental health disorders,^21^ and AOD dependence commonly co‐occurs with mental health issues, there are distinct differences between the two sectors in scope, culture, philosophy, workforces and intervention approaches.^22^ Many people who use alcohol and other drugs do not experience mental ill health, and many experience, or are at increased risk of experiencing, harms or problems beyond dependence (e.g., contact with the criminal justice system).^22^

People who use drugs (whether co‐occurring with mental health issues or not) report specific experiences of service use that may be different from other people's experiences, such as those with depression or anxiety with no co‐occurring illicit drug use. Studies have, for instance, examined the particular role of judgement, stigma and marginalisation on the service‐use experiences of people who use drugs, especially people who use illicit drugs and/or who inject drugs, and people accessing harm reduction services.^23^, ^24^ In addition, the service use experiences of people who use drugs may be shaped by an increased risk of experiencing violence, and contact with the police and criminal justice, welfare and child protection systems.^24^, ^25^ Being dependent on a drug may be seen by others as a personal choice or a moral failing, with people who use drugs being seen as dangerous and deserving of blame.^22^, ^26^ PRMs that are heavily weighted with mental health questions may be less relevant to experiences of AOD treatment and may miss elements of service experience that are particular to people who are dependent on alcohol and other drugs. This warrants the development of dedicated PRMs for use specifically for AOD services.

### 1.4 Measures that are meaningful to the lives and experiences of people who use AODs

The most significant recent criticism of AOD satisfaction and experience measures has been that they do not reflect the values, needs and actual experiences of AOD service users.^9^, ^27^, ^28^, ^29^, ^30^, ^31^ Instead, they reflect the understandings, priorities and needs of service providers, policy makers and researchers.^9^ This has been attributed to the low levels of involvement by AOD service users in processes to develop PRMs of satisfaction and experience.^9^, ^28^, ^32^ The reliability and utility of PRMs of both satisfaction and experience would be significantly improved by the involvement of people who use AOD services in their development.^28^, ^33^

While this criticism has been made, the extent to which AOD service users have (or have not) been included in the process of developing PRMs of satisfaction or experience has not been systematically documented. A greater understanding of how service users have been engaged in this process will inform how the AOD sector is able to improve PRMs of experience, and consequently to improve person‐centred care. A scoping review is most appropriate to this topic to map and synthesise the available evidence, and to identify the key factors and gaps in understanding related to how AOD service users are involved in the development of measures of satisfaction and experience.^34^

The following aim was developed to guide the literature search: How and to what extent have people using AOD services been involved in the development of PRMs designed to assess their satisfaction and/or experiences of AOD treatment and harm reduction services? In pursuing this aim, specific questions included:

1. Which PRMs of satisfaction and/or experiences have been developed specifically for use in AOD treatment and harm reduction services?
2. Across which AOD treatment and harm reduction intervention settings and in which contexts have these been developed?
3. To what extent have the psychometric properties of these measures been assessed?
4. How have people who use drugs been involved in the development of these measures?

## 2 METHODS

The protocol for this review was broadly guided by the Preferred Reporting Items for Systematic Reviews and Meta‐Analysis Protocols for Scoping Reviews.^35^ Searches of the *Joanna Briggs Institute Evidence Synthesis Journal*, Open Science Framework, Figshare and the Cochrane database indicated that no scoping review had been conducted on this topic. Unless contextually necessary, this review purposefully applies advice from guides on the appropriate use of destigmatizing language when writing about people who use drugs, for example, the use of the word dependence instead of disorder.^36^

### 2.1 Search strategy

The search was conducted in early March 2022, using a search strategy developed with advice from a Library Research Consultant, and detailed in Supporting Information: Table 1. The search included published and grey literature, and was limited to full‐text studies published since 1 January 2000, and to English language publications. The final search results were exported into EndNote and duplicates were removed using a systematic protocol.^37^

### 2.2 Review of sources: Stage 1

Papers or reports included in the review described the development and/or implementation of a multiple‐item measure of patient‐reported experience or satisfaction with service delivery by programmes for AOD‐specific treatment (e.g., residential rehabilitation, opioid maintenance therapy [OMT]), or for AOD‐related harm reduction (e.g., needle and syringe programmes). Papers were excluded where people experiencing mental illness were the only, or primary, target group, even where AOD use may have been included as a secondary issue. The review concerns measures that have been developed specifically for people who use alcohol and other drugs, including measures that were developed for both AOD and mental health services at the same time—due to their integration within the same section of the health sector. Supporting Information: Table 2 details the reviews' inclusion and exclusion criteria—these were tested and refined using a subset of papers before finalisation.

Titles remaining after deduplication were reviewed for relevance to these inclusion and exclusion criteria. A large number of papers could be easily excluded as they studied unrelated diseases or conditions (e.g., cancer, asthma, diabetes, dementia), or described reviews or qualitative studies. Smoking‐related titles were excluded—unless they directly concerned people accessing AOD treatment—as, in most countries, nicotine dependence treatment and support are not provided in dedicated treatment services separate from AOD treatment.

The first author then reviewed all remaining papers by abstract using the inclusion and exclusion criteria (Supporting Information: Table 2). A systematic random sampling frame was used to select 10% of abstracts, and these were reviewed by a second author. Where there was any ambiguity based on the content of the abstract, the authors included the paper for full‐text review. Similarly, a full‐text review was conducted by the first author followed by a review of 10% of the papers by a second author. Any discrepancies between the two reviewers were resolved through discussion; no false negatives were identified (i.e., excluded papers that should have been included).

The search was supplemented by a thorough check through the reference lists of the remaining papers; potential additional papers were assessed progressively by title, abstract and full text, and where appropriate included as eligible papers.

### 2.3 Review of sources: Stage 2

All papers identified in stage 1 were grouped according to the PRM of satisfaction or experience that they developed or used; 36 discrete measures were identified across 100 papers. For each discrete measure, the reviewers identified one or two key papers that described the development of the measure and/or tested its psychometric properties. Where they were not reported in the same paper, a second paper was included for analysis. Fifty‐four papers were discarded as not necessary for understanding the identified measures.

For discrete measures, where the method of development of an experience or satisfaction measure was not clearly described in any paper, a thorough internet search was conducted to source documentation that might more completely describe the development of the measure. Where an original article or document describing the development of the measure could not be found or where it was in a non‐English language, the measure (and any paper or papers associated with it) were removed from the review.

### 2.4 Data extraction and synthesis

Two authors independently extracted and recorded the following information from each source of evidence included in the final analysis (i.e., the ‘development’ and/or ‘psychometrics’ paper[s] related to each measure): title; year developed; description of domains; AOD setting; country and context; psychometric properties and whether service users were involved in development. Any discrepancies between the two reviewers were resolved through discussion. Measures were categorised as ‘satisfaction’ or ‘experience’ measures based largely on self‐identification—for example, the name of the measure; or the types of outcomes reported.

#### 2.4.1 Involvement of stakeholders in measure development

In the absence of an existing framework for describing service user involvement in the development of measures of satisfaction or experience, a framework was developed for this scoping review. The framework includes stages and corresponding activities in part adapted from two sources that specifically describe the involvement of service users in the development of patient‐reported *outcome* measures (PROM): a scoping review across various health sectors by Wiering et al.^15^; and a methodology for PROM development involving people experiencing mental ill‐health described by the Service User Research Enterprise.^38^ The framework was further refined with the addition of activities identified in the articles that are included in this scoping review (e.g., the inclusion of service user input into interpreting data and naming domains).^39^

The resulting framework includes five stages of the measure‐development process: (1) research activities and/or governance processes; (2) determining experience dimensions or themes; (3) item development; (4) survey refinement and finalisation and (5) testing for comprehensibility and/or acceptability (see Table 1). Each stage includes activities that describe different types of involvement of service users and/or other stakeholders in the development of measures of satisfaction and/or experience. The level of detail of this framework matches the detail of measure development described in the majority of studies in this review.

**Table 1:** Activities describing involvement by AOD service users and other stakeholders in each stage of the development of patient‐reported measures of satisfaction and experience.

Stage of development	Activities describing involvement by AOD service users and other stakeholders in each stage	Article references (measures with multiple articles are only noted once)	Proportion
(1) Research activities and/or governance processes Describes how and who was involved in governance processes such as Advisory Groups, and in the research process (e.g., data collection, analysis)	1a. AOD service users and/or representatives of peer‐based consumer organisations are members of an Advisory Group or have a formal input mechanism to Group	[31, 40, 41, 42]	4/30
	1b. Formal research partnership with peer‐based consumer organisations (e.g., memorandum of understanding; partnership agreement; formal collaboration)	[16, 29, 41, 43, 44, 45]	6/30
	1c. Peer researchers (i.e., people with lived experience of accessing AOD services) are leading the research, or have formal roles in the research team and are involved in data analysis/interpretation	[16, 42]	2/30
	1d. Peer researchers (i.e., people with lived experience of accessing AOD services) have formal roles in the research team and are involved at least in data collection; and/or assistance in data collection provided by the peer‐based organisation	[16, 41, 44, 46]	4/30
	1e. AOD service users' involvement in governance processes and/or research activities (other than as participants) is not reported or adequately described	[30, 47, 48, 49, 50, 51, 52, 53, 54, 55, 56, 57, 58, 59, 60, 61, 62, 63, 64, 65]	20/30
(2) Determining experience dimensions or themes Describes how and who was involved in determining which experience dimensions or themes will be measured	2a. Involvement by AOD service users in determining the content of topic guides for focus groups/interviews	[29, 42]	2/30
	2b. AOD service users participate to identify dimensions or themes of experiences of service use (e.g., through interviews, focus groups, or Advisory Group mechanisms)	[29, 30, 31, 40, 41, 42, 44, 48, 49, 50, 52, 66]	12/30
	2c. AOD service users provide input into interpretation of dimensions or themes and/or naming of domains	[42]	1/30
	2d. Input from peer‐based consumer groups into dimension or theme development	[16, 29, 43]	3/30
	2e. Dimensions/themes of experience are derived from the literature, theory, previously validated scales, or other experts whose peer status is not confirmed	[16, 29, 30, 31, 40, 41, 43, 45, 46, 47, 49, 50, 51, 52, 53, 56, 58, 59, 60, 62, 63, 64, 65]	23/30
	2f. Method for determining experience dimensions/themes is not reported or adequately described	[54, 55, 57, 61]	4/30
(3) Item development Describes how and who was involved in developing items corresponding to each of the dimensions or themes	3a. AOD service users involved in generating items against each theme (e.g., through focus group discussions, interviews, or other mechanisms, such as advisory structures)	[29, 31, 40, 42, 46]	5/30
	3b. Peer‐based consumer groups involved in generating items against each theme	[16, 29, 31, 43, 45]	5/30
	3c. Items are developed from literature, theory, previously validated scales, or other experts whose peer status is not confirmed	[16, 30, 31, 40, 41, 42, 43, 46, 47, 49, 50, 51, 52, 53, 56, 58, 59, 61, 62, 63, 64, 65]	22/30
	3d. Method for item development is not reported or adequately described	[44, 48, 54, 55, 57, 60]	6/30
(4) Survey refinement and finalisation Describes how and who was involved in reviewing and finalising the draft survey	4a. Review of, and feedback provided, on one or more versions of the draft survey(s) by AOD service users and/or peer‐based consumer groups (including through Advisory structures)	[29, 30, 31, 40, 46]	5/30
	4b. Review of, and feedback provided, on one or more versions of the draft survey(s) by stakeholders (other than researchers), such as service providers, or policymakers	[29, 30, 31, 43, 51, 52, 58]	7/30
	4c. Only researchers involved in the review, or method for survey refinement and finalisation are not reported or adequately described	[16, 41, 42, 44, 45, 47, 48, 49, 50, 53, 54, 55, 56, 57, 59, 60, 61, 62, 63, 64, 65]	21/30
(5) Testing for comprehensibility and/or acceptability Describes how and who was involved in testing the questionnaire to ensure that it is understandable and interpreted correctly	5a. Questionnaire tested with formalised process of testing for comprehension and clarity with AOD service users (e.g., cognitive interviews)	[41, 42, 50, 51]	4/30
	5b. Questionnaire tested with AOD service users or peer‐based consumer groups with opportunities given to provide feedback	[29, 30, 40, 45]	4/30
	5c. Questionnaire pilot tested with AOD service users with no formal mechanism for feedback reported	[31, 46, 47, 49, 55]	5/30
	5d. Questionnaire pilot tested with other stakeholders (e.g., service providers) with or without a formal mechanism for feedback	[41, 43, 46, 52]	4/30
	5e. Method for testing for comprehensibility is not reported or adequately described	[16, 44, 48, 53, 54, 56, 57, 58, 59, 60, 61, 62, 63, 64, 65]	15/30

*Note*: Shaded rows indicate activities where AOD service users were involved in that stage of measure development; unshaded rows indicate no, ‘not reported’, or inadequately described AOD service user involvement.

Abbreviation: AOD, alcohol and other drugs.

For each identified measure (*n* = 30), text describing its development was extracted, and this was then assessed against each stage described in the framework. Consequently, for each measure, one or more activities were assigned to describe the type of involvement of service users and other stakeholders in each stage. Most studies included in this review do not contain sufficient detail to reliably assess and judge the degree or quality of service users' involvement in each activity or process. Values have, therefore, neither been assigned to the degree of service users' involvement (e.g., ‘consultation’, ‘collaboration’, ‘user‐led’),^67^ nor to the quality. The framework focuses on whether service user involvement in each stage was reported or not, and what type of activity was involved (e.g., Advisory Groups, focus group discussions, peer researchers). Within each activity describing service user involvement (the shaded rows in Table 1), the degree of involvement could, therefore, range from a simply consultative role to user‐led participation. Studies that involved multiple stakeholders or methods in their development may be noted in multiple activities within the same stage of development—for example, a paper that described both focus group discussions with service users and reference to other experts in the determination of dimensions of experience would be noted in both rows.

The recording of service user involvement was determined by the development process documented in the included papers. If authors did not report aspects of service user involvement—or did not report service user involvement at all—then these were not recorded in the review. Similarly, if they did not report on the reliability or validity of surveys, then this would be noted as ‘not reported’.

## 3 RESULTS

### 3.1 Selection of sources of evidence

**Figure 1:**
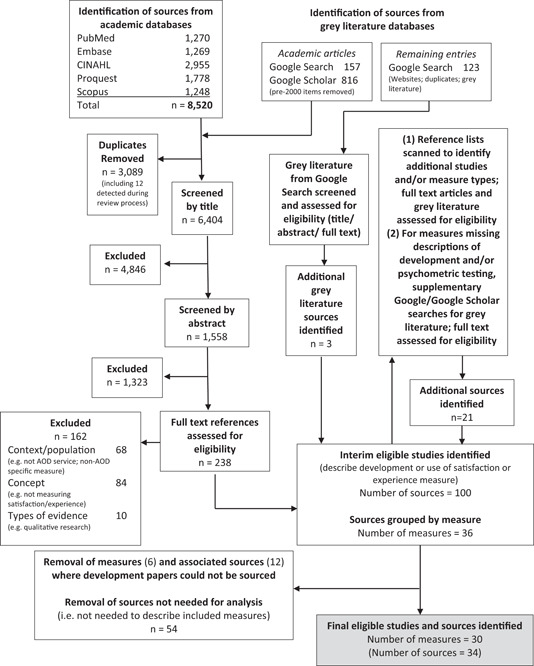
Flow diagram showing the selection of sources of evidence for this review. AOD, alcohol and other drug.

Figure 1 shows the flow diagram for the selection of sources of evidence for this review. Following the abstract and full‐text screening, scanning of reference lists and supplementary searches (as described above), 100 sources were identified across 36 measures. Six measures and their 12 associated papers were removed as articles or reports describing their development could not be located (four measures) or were not in English (two measures). After the removal of 54 sources that were not needed for the analysis (i.e., they were not needed to describe the included measures), there were 30 satisfaction/experience measures (with 34 papers related to them) included in the analysis. For four measures, a second paper was included describing the testing of psychometric properties,^39^, ^68^, ^69^ or an additional component of the measure's development.^66^

### 3.2 Measure characteristics

Of the 30 measures identified in this review, 23 (77%) were satisfaction measures, and 7 (23%) were experience measures—although some of these experience measures included ‘satisfaction’ as an element of the survey. Supporting Information: Tables 3a and 3b describe the characteristics of each of these measures.

#### 3.2.1 Trend over time

Satisfaction‐focused measures dominated the early years of this review; all 17 of the measures developed between 1995 and 2007 were satisfaction focused. All identified experience measures were developed after 2008, compared to only 6 of the 23 satisfaction measures. Similarly, the number of measures reporting AOD service user involvement in their development increased over time, from one out of six measures in 1995–1999 to almost all measures in 2015–2019 (seven of eight).

#### 3.2.2 Treatment settings and context

Seventeen measures were developed specifically for use in a single service type, with 13 measures developed for use across multiple programme types. OMT programmes were the most widely represented treatment setting. Sixteen measures were developed for use in OMT settings: nine for exclusive use in OMT programmes; and seven for a range of service types that included OMT. Most (26) were measures only intended for use in AOD‐specific *treatment* settings, including OMT. Only four measures included one or more *harm reduction*‐specific intervention settings: needle syringe programmes (2)^43^, ^47^; drop‐in education and information programmes (3)^29^, ^43^, ^47^; and a drug consumption room (1).^48^

Twelve measures (seven satisfaction, five experience) were specifically developed to assess across state‐ or national‐level AOD service systems (as opposed to within a single service or organisation), with four of these used specifically for jurisdiction‐wide OMT programmes, and nine measuring across a mix of different AOD service types. However, only two of these—the NTA survey^47^ and the SUSOS^43^—measured across both AOD‐specific treatment *and* harm reduction service types. Both were satisfaction surveys and only one^43^ involved service users in its development.

#### 3.2.3 Psychometric properties

Two‐thirds of studies (20) included in this review undertook some form of psychometric testing of the developed measures. Five measures that were developed with service user involvement did not assess and/or explicitly report on their psychometric properties.^29^, ^44^, ^45^, ^46^, ^48^ Almost half (13) of the measures—including seven developed with service user involvement^31^, ^39^, ^40^, ^49^, ^50^, ^51^, ^69^—reported undertaking some form of analysis of the underlying scale structure and constructs of the measures (using exploratory factor analysis, confirmatory factor analysis or principal component analysis). Eight studies explicitly reported that they confirmed the face (7) and/or content (2) validity of their measures,^30^, ^41^, ^42^, ^49^, ^51^, ^52^, ^53^, ^68^ with six of these reporting involvement of AOD service users in their development.

### 3.3 Involvement by AOD service users

Table 1 shows the involvement of AOD service users in each stage of the development of the PRMs of satisfaction and experience, and outlines how each stage was described in the 30 measures included in this scoping review. Supporting Information: Table 4 shows the assessment of each measure against each stage described in the framework (see Section Methods, 2 for further details). The shaded rows indicate activities where AOD service users were involved in that stage of measure development; the unshaded rows indicate no, ‘not reported’, or inadequately described AOD service user involvement (although other stakeholders may have been involved). For four of the five stages—all except ‘(2) Determining experience dimensions or themes’—at least two‐thirds of the measures had either no involvement of AOD service users or did not report such involvement.

Sixteen of the included measures reported some level of involvement by people accessing AOD services in their development: 9 of 23 satisfaction surveys; and all 7 of the experience measures. The most common activity that involved AOD service users was ‘AOD service users participate to identify dimensions or themes of experiences of service use (2b)’—described for 12 measures (Table 1). This activity involved listening to and understanding the viewpoints, opinions, and experiences of AOD service users through focus groups, interviews or Advisory Group structures to elicit the range of concepts and dimensions of experience that were translated into the items and constructs used in experience measures. Most of these studies reported using qualitative findings to inform, clarify and expand domains that had previously been identified through the literature or existing measures (i.e., a deductive approach). This scoping review identified one measure^39^, ^42^ that used an inductive approach to identify themes of service user experience and then worked collaboratively with service users to translate these to experience domains through a process of survey testing, factor analysis and engagement in interpretation processes. Other commonly described activities were: ‘formal research partnership with peer‐based consumer organisations’ (1b—described for six measures); ‘AOD service users involved in generating items against each theme’ (3a—five measures); ‘peer‐based consumer groups involved in generating items against each theme’ (3b—five measures) and ‘review of, and feedback provided, on one or more versions of the draft survey(s) by AOD service users and/or peer‐based consumer groups’ (4a—five measures).

## 4 DISCUSSION

This review confirms observations in the literature, that satisfaction surveys have dominated in the AOD sector, and until recently have been more widely used than experience measures to assess the quality of person‐centred care in AOD services.^8^, ^9^ Researchers have noted the relative absence of AOD‐specific experience measures and their low level of use in AOD services research and evaluation.^8^, ^32^ Measuring experience, beyond ‘satisfaction’, captures and describes the quality of the care received, allowing for greater discrimination from patient expectations, and linking more clearly to priorities for service quality improvement.^6^, ^9^, ^70^ As in the health sector generally, the utility of AOD PRMs to quality improvement is limited by the design of these measures, the interpretation of data coming from them and the actual translation and implementation of this data to inform interventions that improve AOD treatment or practice.^5^, ^32^, ^71^

As far as the authors can determine, this scoping review is unique in examining the involvement of service users in the development of patient‐reported *experience* measures. The review has included setting out a framework with stages of experience‐measure development, and activities within these that describe opportunities for service user involvement at each stage. The stages of development each have different purposes and potential mechanisms for service user involvement.

Assessing against this framework, the review has found that just over half of the satisfaction or experience measures reported involve AOD service users in some way in their development. The reporting of such involvement appears to have increased over time, as has a preference for the inclusion of experience, rather than solely satisfaction‐type, measures. This suggests that service user involvement in measure development is a defining feature of experience measures.

Despite this increase over time, for most measures, the involvement of service users was limited; only six of the measures—two satisfaction,^16^, ^41^ four experience^29^, ^31^, ^40^, ^42^—were assessed as involving AOD service users in five or more measure‐development activities (out of 13). Importantly, the framework used in this study has proved a useful tool for assessing the presence or absence of service user involvement for each activity type and can inform other reviews of patient‐reported experience measure development. There is scope for further refinement of the framework to reflect consensus on what defines the *quality* of service user involvement in each activity of measure development. Such refinement will identify the actual extent to which service users participate in the development of experience measures and, in turn, identify experience measures that best reflect service user perspectives and that measure domains that are genuinely relevant to AOD service users.^32^

Several studies in this review highlight ways to respectfully engage with, and support the participation of, people who access AOD services in research processes. The review identifies two experience measures with likely high quality of involvement by AOD service users in their development.^29^, ^39^, ^42^ Both include intensive and formalised collaborative processes of service user involvement through advisory structures, expert groups and research activities, and one also established a formal intensive engagement process to include service users in the analysis and reporting.^39^

Other studies in the review also report engagement through the use of specific and innovative approaches including: dialogue, visually‐based methods and culturally relevant techniques, such as talking circles, graphic recording and flash cards.^29^, ^30^ Not only may these approaches be more culturally relevant for some participants, but they also address issues of literacy and capacity for engagement by people experiencing cognitive issues or chaotic lives. Studies also suggest supporting participation by addressing the potential inequality of resourcing available to people accessing AOD services; this includes, for example, offering payments, providing training and taking flexible approaches to engagement.^39^ In the absence of such approaches, there may be real barriers to recruitment and engagement of service users in the research process, affecting the representativeness of participants in measure design and their utility in practice.

The benefits of involving service users in the development of experience measures are clear to researchers undertaking these survey development processes.^29^, ^30^, ^39^ Such involvement enables the identification of AOD‐specific and locally‐relevant dimensions of care to inform meaningful survey items to identify priority areas for improvement and action.^39^ Intensive input from the service user perspective will lead to more patient‐centred measures^32^ as these processes: uncover specific and relevant unmet service needs, as well as barriers and facilitators to accessing care^29^; identify and help frame questions of particular importance, such as cultural safety or stigma^29^ and improve the structure, language and clarity of the questionnaire. In short, as articulated by one research team, collaborating with service users ‘helped ensure our questionnaire captured constructs that were meaningful to participants and that questions were posed in clear and appropriate language’.^29^,^p.21^

High confidence in tool reliability and validity corresponds to greater confidence that survey findings come closer to reflecting the reality of service experience.^6^ This in turn gives greater confidence that higher satisfaction and better experiences are indeed associated with improved substance use outcomes, including lower severity of dependence and levels of substance use, reduced frequency of substance use and higher odds of abstinence.^8^ Fewer than half of the measures identified in this review reported undertaking some form of analysis of the underlying scale structure and measure constructs, and fewer than one‐third explicitly reported on their face and/or content validity. Improved reporting on the psychometric properties of PRMs of satisfaction or experience will assist AOD researchers and practitioners to select validated tools and to have greater confidence in findings and how these relate to clinical outcomes and service quality improvement.

A gap identified through this scoping review is validated experience measures developed for, and tested in, settings specifically designed to deliver harm reduction interventions—for example, needle and syringe programmes, supervised consumption rooms, drop‐in services and peer support programmes.^72^ Only four measures have been implemented in harm reduction‐specific‐intervention settings, with three of these implemented alongside other intervention types. PRMs therefore appear to be rarely developed for, or administered within, harm‐reduction settings. This may be because service user–provider interactions in these contexts are characterised by their capacity to be transient and anonymous, facilitating the delivery of interventions that reduce harm even where AOD use may continue. This context may mean the implementation of a service user‐completed survey tool such as a PRM would be more difficult. The absence of experience measures specific to the harm reduction service context may also be explained by the real or perceived stigma that has been noted to inhibit the involvement of people who use drugs in research and quality improvement processes more generally.^73^, ^74^

Further, PRMs are commonly used in discrete health service contexts (e.g., a specific ward or health programme). However, they are increasingly also being used at a jurisdictional level to measure performance and to drive improvements in the quality and safety of value‐based and person‐centred care across healthcare sectors and health systems.^10^, ^75^, ^76^ Satisfaction and experience measures developed for use across jurisdictional AOD service systems appear to be largely limited to specific intervention types, in particular, AOD treatment interventions such as OMT^55^, ^68^; or residential rehabilitation and/or outpatient treatment.^31^, ^50^, ^51^ Further research and developmental work are needed to develop experience measures for use across the entire AOD service system that includes harm reduction‐specific services along with other AOD intervention types.

AOD service user participation in a range of policy‐making, research, programming and practice has been linked to relevant, equitable and effective AOD treatment and support.^77^ Partnering with people who use AOD in the research process to design, develop and implement PRMs, challenges dominant knowledge paradigms, addresses power imbalances and signals a commitment to social justice and person‐centred care by AOD services.^28^ Further, involving service users in the design and development of PRMs is likely to improve the validity (including face and content validity) and reliability of these measures, and so improve the quality of the evidence towards AOD service quality improvement and sector planning.^15^, ^28^, ^29^

### 4.1 Limitations of the review

Limitations of this review include the possibility that relevant studies were not identified in the search process, or were excluded by language bias (as only English‐language publications were included) or publication bias (e.g., measures developed by community‐based organisation that were not formally published). Further, the recording of service user involvement and/or reporting on measure validity or reliability was determined by the processes documented in the included papers. Where these were absent or incompletely documented, measures may have been miscategorized within the framework developed for the review.

## 5 CONCLUSION

This review has found 16 (out of 30) PRMs of satisfaction or experience identified using a systematic and comprehensive search of the literature have involved people who use drugs in their development, although with varying intensities of involvement. It confirms the increased use over time of experience, as opposed to satisfaction, measures in the AOD sector and increased reporting of AOD service user involvement in the development of measures. Most measures were developed for use in AOD‐specific treatment settings, rather than harm reduction‐specific intervention settings. There were no experience measures identified that were both developed for use in AOD harm reduction settings and that involved service users in their development.

The review has identified several areas that would benefit from further development in this field. This includes: developing experience measures suitable for use in harm reduction settings; and developing measures of service user experience across entire AOD systems, including various AOD treatment and harm reduction settings. More specifically, a high‐quality evaluation of person‐centred AOD treatment and support is likely to be improved by increasing the intensity and quality of involvement of AOD service users in such measure development. To this end, improving consensus on, and reporting of, the quality of involvement by AOD service users should be a priority, and this could be achieved by expanding the framework proposed in this scoping review to reflect quality, not just the presence or absence, of service user involvement. Further, formally assessing and reporting the face and content validity of experience measures will provide more robust evidence of the value of involving AOD service users in the development of these measures.

## AUTHOR CONTRIBUTIONS

All authors contributed to the conceptualisation of the review and framework. Anke E. van der Sterren wrote the protocol and performed the search, with Sally Nathan, Devin Bowles and Patrick Rawstorne critically reviewing the protocol. Anke E. van der Sterren and Elisabeth Yarbakhsh screened the citations and extracted the data. Anke E. van der Sterren conducted the analysis and interpretation against the framework, with Sally Nathan, Devin Bowles and Patrick Rawstorne reviewing the analysis. Anke E. van der Sterren wrote the manuscript, with all of the remaining authors critically reviewing the draft manuscript. Chris Gough provided specific advice on the framing of the review and the appropriate use of language and terminology. All authors approved the final document.

## CONFLICT OF INTEREST STATEMENT

The authors declare no conflict of interest.

## Supporting information

Supporting information.

Supporting information.

Supporting information.

Supporting information.

## Data Availability

The data that support the findings of this study are available in the Supporting Information: Material of this article or from the corresponding author upon reasonable request.
