# Supplementary material for: Involvement of people who use alcohol and other drug services in the development of patient‐reported measures of experience: A scoping review
Source: Health Expect. 2023 Jul 29;26(6):2151–63. doi: 10.1111/hex.13829 (PMC10632652; doi:10.1111/hex.13829)
Supplement: Supplementary file 1 — Supporting information. [file HEX-26--s004.docx]

# Supplementary Table 1: Search Strategy, showing primary search strategy (PubMed), and converted search strategy syntax for each other database, and method for grey literature search

Filters for all searches:

- Language: English
- Date range: from 1 January 2000

| **Database** | **Equivalent terms** | | | | | | | | |
| --- | --- | --- | --- | --- | --- | --- | --- | --- | --- |
|  | **COMBINED USING AND** | | | | | | | | |
|  | **COMBINED USING OR** | | | | **COMBINED USING OR** | | | | |
| 1. PubMed (primary search strategy) | "Substance Abuse Treatment Centers"[Mesh] | "Drug Users"[Mesh] | "Substance-Related Disorders"[Mesh] | ((("alcohol and drug*"[Text Word]) OR ("alcohol and other drug*"[Text Word])) OR ("drug and alcohol"[Text Word])) OR ("drugs and alcohol"[Text Word]) | "Patient Satisfaction"[Mesh] | "Patient-Centered Care"[Mesh] | "Patient Reported Outcome Measures"[Mesh]  “patient reported experience measure*”[Text Word]  “perception of care”[Text Word] | ((("patient satisfaction"[Text Word]) OR ("client satisfaction"[Text Word])) OR ("consumer satisfaction"[Text Word])) OR ("service user satisfaction"[Text Word]) | ((("patient experience*"[Text Word]) OR ("client experience*"[Text Word])) OR ("consumer experience*"[Text Word])) OR ("service user experience*"[Text Word]) |
| The primary search strategy was developed in PubMed, and then mapped onto search strategy syntax for: EMBASE; CINAHL; Scopus; and ProQuest. | | | | | | | | | |
| 1. EMBASE (Ovid) | exp drug dependence treatment/  substance abuse treatment cent*.mp. | drug user*.mp. | exp drug dependence/ep, pc, rh, th [Epidemiology, Prevention, Rehabilitation, Therapy]  substance related disorder*.mp. | (alcohol adj2 drug*).mp. | satisfaction/ or patient satisfaction/ | patient centered care.mp  Patient centred care.mp. | patient-reported outcome/  patient reported outcome measure*.mp  patient reported experience measure*.mp  perception of care.mp. | (patient satisfaction or client satisfaction or consumer satisfaction or service user satisfaction).mp. | (patient experience* or client experience* or consumer experience* or service user experience*).mp. |
| 1. CINAHL | (MH "Substance Use Rehabilitation Programs+")  "substance abuse treatment cent*" | (MH "Substance Abusers+") | (MH "Substance Use Disorders+")  (MH "Substance Dependence+")  “substance related disorder*” | alcohol N2 drug* | (MH "Patient Satisfaction+")  (MH "Consumer Satisfaction") | (MH "Patient Centered Care")  “patient cent* care” | (MH "Patient-Reported Outcomes")  (MH "Outcome Assessment")  “patient reported experience measure*”  “perception of care” | "patient satisfaction" OR "client satisfaction" OR "consumer satisfaction" OR "service user satisfaction" | "patient experience*" OR "client experience*" OR "consumer experience*" OR "service user experience*" |
| 1. ProQuest (used APA Thesaurus of Psuchological Index Terms – Summer 2021; MeSH 2022 Thesaurus; ProQuest Thesarus) | MAINSUBJECT.EXACT("Substance Use Treatment")  MAINSUBJECT.EXACT("Substance abuse treatment")  MAINSUBJECT.EXACT("Addiction Treatment")  MAINSUBJECT.EXACT("Needle exchange programs")  MESH.EXACT("Substance Abuse Treatment Centers")  MESH.EXACT("Needle-Exchange Programs") | MESH.EXACT("Drug Users") | MAINSUBJECT.EXACT("Drug abuse")  MAINSUBJECT.EXACT("Drug addiction")  MAINSUBJECT.EXACT("Drug use")  MAINSUBJECT.EXACT("Substance Use Disorder")  MAINSUBJECT.EXACT("Opioid Use Disorder")  MESH.EXACT("Substance-Related Disorders") | ti(alcohol NEAR/2 drug*)  ab(alcohol NEAR/2 drug*) | MAINSUBJECT.EXACT("Patient satisfaction")  MAINSUBJECT.EXACT("Client Satisfaction")  MESH.EXACT("Patient Satisfaction") | MAINSUBJECT.EXACT("Patient Centered Care")  MAINSUBJECT.EXACT("Patient-centered care")  MAINSUBJECT.EXACT("Client Participation")  MESH.EXACT("Patient-Centered Care")  MESH.EXACT("Patient Participation") | MAINSUBJECT.EXACT("Patient Reported Outcome Measures")  MESH.EXACT("Patient Reported Outcome Measures")  ti("patient reported experience measure") OR ab("patient reported experience measure")  ti(“perception of care”)  ab(“perception of care”) | ti("patient satisfaction") OR ti("client satisfaction") OR ti("consumer satisfaction") OR ti("service user satisfaction")  ab("patient satisfaction") OR ab("client satisfaction") OR ab("consumer satisfaction") OR ab("service user satisfaction") | ti("patient experience*") OR ti("client experience*") OR ti("consumer experience*") OR ti("service user experience*")  ab(" patient experience*") OR ab("client experience*") OR ab("consumer experience*") OR ab("service user experience*") |
| 1. Scopus | TITLE-ABS-KEY ( "drug dependence treatment" )  TITLE-ABS-KEY ( "substance abuse treatment centers" ) | TITLE-ABS-KEY ( "drug users" ) | TITLE-ABS-KEY ( "substance related disorders" )  TITLE-ABS-KEY ( "drug dependence" ) | TITLE-ABS-KEY ( alcohol W/2 drug* ) | TITLE-ABS-KEY ( "patient satisfaction" ) | TITLE-ABS-KEY ( "patient centered care" ) | TITLE-ABS-KEY ( "patient reported outcome measures" )  TITLE-ABS-KEY ( "patient reported experience measure" )  TITLE-ABS-KEY  (“perception of care”) | TITLE-ABS-KEY ( "client satisfaction" )  TITLE-ABS-KEY ( "consumer satisfaction" )  TITLE-ABS-KEY ( "service user satisfaction" ) | TITLE-ABS-KEY ( "patient experience" )  TITLE-ABS-KEY ( "client experience" )  TITLE-ABS-KEY ( "consumer experience" )  TITLE-ABS-KEY ( "service user experience" ) |
| Grey literature search: To capture grey literature, a secondary search approach was undertaken using Google Search and Google Scholar.^1, 2^ To minimise the personalisation of results from the search algorithm of these search engines, the browser’s search history data was deleted, and all Google accounts were logged out prior to the search.^2^ The results have been specifically documented for future reference, as the search algorithms in Google mean that these searches are not replicable.^2^  The search strategies were consistent with the terms used for the academic databases, and were adjusted for each search engine to maximise the return of articles, reports and webpages that were most relevant to the review (see Supplementary Table 1). The results were examined systematically up to, and including, page 30 (300 results), as recommended by Haddaway et al.^1^ For Google Search, where links led to academic articles, these were automatically exported into Endnote; where links were directed to organisational or government websites, a further search was conducted of publications appearing on these sites—any reports or papers that were identified, were reviewed as described below. | | | | | | | | | |
| 1. [Google](https://www.google.com.au/advanced_search) Search |  |  | alcohol\|drug\| addiction |  |  |  | ”perception of care” | "patient satisfaction”  “client satisfaction"  "service user satisfaction"  "consumer satisfaction" | "patient experience"  "client experience"  "service user experience"  "consumer experience" |
| 1. [Google Scholar](https://scholar.google.com/)* |  |  | "alcohol and drug"\|"drug and alcohol"\|addiction |  |  |  | ”perception of care” | "patient satisfaction"\|"client satisfaction"\|"service user satisfaction"\|consumer satisfaction” | “patient experience"\|"client experience"\|"service user experience”\|”consumer experience” |

* To ensure maximum coverage, and to manage the character length and search string limitations, three searches were undertaken in Google Scholar to allow for variations of the use of “alcohol and drug”, “drug and alcohol” and addiction—in combination with “patient (and synonyms) experience”, “patient (and synonyms) satisfaction”, and “perception of care”. This enabled the search to be narrowed to AOD-related content, rather than returning all articles related to drugs (including medications) of any kind.

References

1. Haddaway NR, Collins AM, Coughlin D, et al. The role of Google Scholar in evidence reviews and its applicability to grey literature searching. *PloS one* 2015; 10: e0138237.

2. Piasecki J, Waligora M and Dranseika V. Google search as an additional source in systematic reviews. *Science and engineering ethics* 2018; 24: 809-810.
