# Supplementary material for: Involvement of people who use alcohol and other drug services in the development of patient‐reported measures of experience: A scoping review
Source: Health Expect. 2023 Jul 29;26(6):2151–63. doi: 10.1111/hex.13829 (PMC10632652; doi:10.1111/hex.13829)
Supplement: Supplementary file 2 — Supporting information. [file HEX-26--s003.docx]

**Supplementary Table 2: Details of inclusion and exclusion criteria for the scoping review**

|  | **Inclusion criteria** | **Exclusion criteria** |
| --- | --- | --- |
| **Population** | 1. Patient-reported measures developed specifically for use with people who use alcohol and other drugs who are seeking treatment or services for AOD dependence or for AOD-related harm reduction. 2. People experiencing dependence; alcohol and all drugs (including prescribed drugs) in scope. | 1. Nicotine dependence, unless co-occurring with AOD dependence. 2. People experiencing mental ill-health were the only, or primary, target group, even where AOD use may have been included as a secondary issue. 3. AOD workers or service providers were target participants of measure |
| **Concept** | 1. The primary focus of the measure was experience or satisfaction; OR 2. Where measuring experience or satisfaction was a component, or secondary consideration in the study/project, but where more than one dimension or concept of experience or satisfaction was being measured. Examples of these dimensions or concepts were: perceptions of staff; facilities; waiting times; and safety. 3. Described satisfaction/experience measures that were: primarily for the purposes of measuring a specific instance of treatment access; comprised of closed-ended questions (e.g. yes/no; Likert scales); and patient-completed (either personally, or by interview). 4. Sources related to experience or satisfaction with service delivery by programs or services. | 1. Sources reporting measures that used a single question to measure satisfaction, or those that focused exclusively on outcomes. 2. Sources reported using generic feedback surveys or reported exclusively on satisfaction/experience with specific activities, interventions or program components, for example satisfaction/experience with: an online assessment tool or information materials; a specific treatment approach within a program (e.g. comparing satisfaction with different intensities of counselling); or satisfaction/experience with specific medications used in AOD treatment. |
| **Context** | 1. Settings for survey design and/or delivery were primarily alcohol and other drug treatment and/or harm reduction services. This included services where people who use drugs access:  - treatment interventions for AOD dependence—including, residential rehabilitation, in-patient or community withdrawal, day programs, counselling, opioid maintenance therapy (including through primary care) - harm reduction services—including needle and syringe programs, peer support services, and drug consumption rooms  1. Measures that have been developed specifically for people who use alcohol and other drugs. 2. Studies describing or using adaptations of measures from other sectors only if there was substantial development work to make them AOD-specific (i.e. adaptations involved more than changing wording from mental health- to AOD-relevant terminology). 3. Measures that were developed for both AOD and mental health services at the same time—due to their integration within the same section of the health sector. | 1. Primary care and services delivering blood borne virus treatment, except where primary care services were delivering opioid maintenance therapy, or other specific AOD treatment or harm reduction services. 2. Measures that have been developed for service users of generic health services or only for mental health services, not specifically for use with people using AOD services. |
| **Types of evidence** | 1. Full papers or reports written in English. | 1. Sources where: only the abstracts were available, including conference abstracts; a full article could not be located or accessed; and where the papers were reviews, commentaries, letters to the editor, or newspaper articles. Studies exclusively using qualitative research methods, unless they were specifically related to the development of a survey measure. |
