# Supplementary material for: Involvement of people who use alcohol and other drug services in the development of patient‐reported measures of experience: A scoping review
Source: Health Expect. 2023 Jul 29;26(6):2151–63. doi: 10.1111/hex.13829 (PMC10632652; doi:10.1111/hex.13829)
Supplement: Supplementary file 3 — Supporting information. [file HEX-26--s001.docx]

**Supplementary Table 3a: Characteristics of patient-reported measures of satisfaction**

| **Patient-reported measure of satisfaction** | **Year developed** | **Source describing development of the satisfaction measure** | **Dimensions/ domain measured** | **AOD treatment or harm reduction settings** | **Context and location** | **Validation** | **Were service users involved in the development of the measure?** |
| --- | --- | --- | --- | --- | --- | --- | --- |
| Chelsea Arbor Treatment Center Patient Satisfaction Survey Outpatient Detoxification | 1995 | Strobbe, Brower & Galen, 2004^1^ | Satisfaction related to: safety; comfort; information and instructions about medications; availability of the nurse; participation in the day treatment program | Outpatient detoxification (any AOD) | Single service; USA | Not assessed/ reported | No |
| Consumer Satisfaction Survey for people accessing opioid maintenance therapy | 2005 | Madden et al., 2008^2^ | Demographics; treatment history; clinical practice; global treatment satisfaction; relations with case managers and prescribers; consumer involvement in treatment planning; satisfaction with key domains of case management; and satisfaction with aspects of service delivery at the clinic | Opioid maintenance therapy | 9 services, single state-level jurisdiction; Australia | Internal consistency | Yes |
| EQUATOR (European Quality and Audit of Opioid Treatment) - based on Project IMPROVE survey | Around 2009 | Stover 2011^3^; Fischer & Stover 2012^4^ | demographics; motivation/goals; access; treatment; information; prison; outcomes' satisfaction | Opioid maintenance therapy | Opioid substitution treatment, multiple European countries (Austria, Denmark, France, Germany, Greece, Italy, Norway, Portugal, Sweden, UK); also used in Switzerland and for multiple Asian country-study | face validity; internal consistency; criterion validity; retest reliability | No |
| Eurasian Harm Reduction Association survey | 2019 | EHRA 2020^5^ | satisfaction with services in general, assessment of (and satisfaction with) physical setting, quality of medical care, and social and psychological support | Opioid maintenance therapy | 3 funded OMT sites, 1 secondary site, 11 primary health-care sites; Ukraine | Not assessed/ reported | Yes |
| Evaluation of Treatment (EOT) measure | 1997 | Kirouac, Witkiewitz & Donovan, 2016^6^ | General satisfaction; dealing with urges; therapist involvement; learning skills; self-growth; mutual help engagement | Alcohol treatment in-patient and outpatient settings (no other drugs; people using other drugs were excluded) | Multiple services across 10 state-level jurisdictions; USA | Factor analysis/construct validity; internal consistency | No |
| Global Appraisal of Individual Needs (GAIN) subscales: Treatment Satisfaction Index (TSI)/ Treatment Satisfaction Scale (TxSS) | Unknown; possibly late 1990s with modifications in early 2000s | Ives et al., 2012^7^ | Working relationship between treatment staff and client  Opinion of treatment received | AOD treatment services (outpatient and residential-various) | Outpatient, residential; developed in USA for use across multiple states, but since used in multiple countries, including USA, Canada, Brazil, China, Japan, and Mexico | Factor structure/construct validity, internal consistency, test-retest, content validity | No |
| Multilevel Satisfaction Evaluation (MSE) | 2005 | Congia et al., 2012^8^ | Treatment received (satisfaction with different components of treatment provided: medical, nursing, psychological, social and educational); Premises, reception and orientation (evaluation of the suitability of places and structures, hours of opening and providing of different services: pharmacy, laboratory analyses, medical visits, psychological interviews, educational and social interventions; clarity of information and ease of access to services); Multidisciplinariety of intervention (number of different operators (physician, psychologist, etc.) involved in treatment) | Outpatient services providing detoxication, opioid agonists and antagonist-maintenance, counselling, general medical care, specific medical care for HIV-related conditions, psychological-psychiatric care and rehabilitative interventions | Single service with three units (two urban, one suburban), Italy | Internal consistency | No |
| National Treatment Agency for Substance Misuse survey of user satisfaction | 2005 | Best, 2007^9^ | Treatment received; perceived gains from treatment; areas of concern and treatment need; attitudes to treatment (treatment impact, respect, treatment engagement and support, client evaluation of self and treatment (CEST) | Residential drug treatment (inpatient and residential rehabilitation), inpatient/outpatient detoxification, OMT, needle syringe programs, drop-in centres, community drug treatment programs (prescribing, day programs, structured psychosocial interventions) | Multiple services, national; England | Not assessed/ reported | No |
| Patient Feedback Survey | 2004 | Forman et al., 2007^10^ | Therapeutic alliance; group treatment satisfaction; self-reported substance use | Outpatient AOD treatment clinics | 6 services across 5 state-level jurisdictions; USA | Internal consistency | No |
| Patient Satisfaction Survey | Before 2007 | Hogan, Hershey & Ritchey, 2007^11^ | Access to care; general satisfaction; provider conduct; outcome of care; financial aspects of care; availability of care; continuity of care | Opioid maintenance therapy | Single service, USA | Not assessed/ reported | No |
| Perceptions of Care Survey | Around 1998 | Eisen et al., 2002^12^ | information provided to patients, interpersonal aspects of care, continuity/coordination of care, global evaluation of care | Inpatient substance abuse treatment | 14 facilities, USA (northeast, south, midwest, west coast) | Factor analysis (EFA and CFA), internal consistency | No |
| Primary Care Buprenorphine Satisfaction Scale (PCBSS) | Around 2005 | Barry et al., 2007^13^ | Satisfaction ratings of: overall and specific service components; staff expertise, concern and responsiveness; helpfulness of overall and specific treatment components | Opioid replacement therapy and counselling (primary care-based) | Single service; USA | Internal consistency, factor analysis (PCA), criterion/predictive validity | No |
| Rankin Court measure | 2003-04 | Kehoe & Wodak, 2004^14^ | Satisfaction: professionals’ skills and behaviours, access, efficacy, information, overall satisfaction, patients’ input to decision making in health care | Opioid pharmacotherapy clinic | Single service; Australia | Face validity | Yes |
| Scientific Evaluation of Supervised Injecting (SEOSI) questionnaire | Around 2003 | Petrar et al., 2007^15^ | Changes to injecting behaviour, perceived barriers to use of facility, perspectives on improvements, service quality (overall, facility and equipment, reliability and dependability of service provision, staff help, courtesy and respect from staff, trustworthiness of staff, acceptance and caring from staff | Safer injecting facility (Supervised consumption room) | Single location, city, Canada | Not assessed/ reported | Yes |
| Service Users’ Satisfaction and Outcomes Survey (SUSOS)/ Service Users’ Satisfaction Survey (SUSS) | 2009 | Alcohol Tobacco and Other Drug Association ACT [ATODA], 2020^16^ | Demographics; overall satisfaction; attitudes to staff; attitudes to services; characteristics of service attendance; service quality and accessibility; ancillary services; outcomes | Residential rehabilitation, inpatient detoxification, outpatient, day program, peer-based, needle and syringe programs, psychosocial, community based, opioid pharmacotherapy | Most AOD programs in single state-level jurisdiction; Australia | Internal consistency | Yes |
| Ted Noffs Foundation Substance Use Assessment Part C | 2000 | Howard & Arcuri, 2005^17^ | Client perceptions of: action plans, groups, individual counselling and family and carer support groups, journaling, vocational and educational assistance, general reflections on and satisfaction with treatment | Residential treatment | Five services (3 metropolitan; 2 rural; Eastern, Australia | Not assessed/ reported | No |
| Telemedicine-delivered medications for opioid use disorder (t-MOUD) | 2019 | Cole et al., 2021^18^ | Satisfaction with: communication; privacy; patient perceptions; technology utilisation; treatment access | AOD Clinic registered to prescribe medications for opioid use disorder via telehealth | Single service; USA | Not assessed/ reported | No |
| Texas Christian University – Client Evaluation Form (CEF)/ Treatment Engagement Form (TEF) from Client Evaluation of Self and Treatment (CEST) | Early 1990s | Joe et al., 2002^19^ | Treatment needs (help for emotional troubles, need of counselling, education/vocational training, medical care and services); counselling rapport (counsellor respect scale, interactions); program satisfaction (overall, specific aspects of treatment related to expectations, convenience, sufficiency of counselling, program organisation, staff, treatment location); treatment participation (cognitive and behavioural involvement and progress); peer support (internal to program); social support (external to program) | Outpatient, outpatient methadone maintenance treatment, residential settings, therapeutic communities, halfway houses, prison programs | Multiple services in 7 state-level jurisdictions; USA | Internal consistency; construct validity/factor analysis (PCA, CFA) | No |
| Treatment Perceptions Questionnaire (TPQ) | 1997-98 | Marsden, Stewart et al 2000^20^ | Satisfaction: Staff perceptions; program perceptions | In-patient detoxification; outpatient; community methadone maintenance treatment program; community team | Multiple services within a city; England | Content and face validity, structure (EPA), internal consistency, test-retest reliability, discriminant validity | Yes |
| Un-named 19-item satisfaction survey (Norwegian Municipalities) | Around 2017 | Stallvik et al., 2019^21^ | Overall satisfaction and important life areas; satisfaction with practical help; satisfaction with personnel experience | Community-based substance abuse services | Multiple municipalities, Norway | Construct analysis (PCA), internal consistency | Yes |
| Un-named patient satisfaction survey (residential rehabilitation) | 2019 | Dhumal, 2019^22^ | Counsellor skills, programmatic structure (adhering), skill development (personal responsibility), preference/comparison to other programs, general satisfaction | Residential rehabilitation | Single residential rehabilitation service, USA | Face validity, internal consistency | Yes |
| Un-named satisfaction survey for opioid treatment at community pharmacies | 2005 | Lea, Sheridan & Winstock, 2008^23^ | Demographics; satisfaction with pharmacy services and overall treatment satisfaction; dispensing fees and credit; waiting times; contracts; dispensing practices associated with pharmacotherapies | Opioid treatment services at community pharmacies | 50 pharmacies across single state-level jurisdiction; Australia | Not assessed/ reported | Yes |
| Verona Service Satisfaction Scale for methadone-treatment (VSSS-MT) | Unknown, published 2002 | Perez de los Cobos et al., 2002^24^ | Overall satisfaction, professionals’ skills and behaviours, information, access, efficacy, types of intervention  Sub-scales: basic interventions; specific interventions; social worker skills; psychologist skills | Outpatient methadone maintenance treatment | Multiple services in national sample, and single state-level jurisdiction; Spain | Structure (EFA), concurrent validity, internal consistency, test-retest reliability | No |

**Supplementary Table 3b: Characteristics of patient-reported measures of experience**

| **Patient-reported measure of experience** | **Year developed** | **Source describing development of the experience measure** | **Dimensions/ domain measured** | **AOD treatment or harm reduction settings** | **Context and location** | **Validation** | **Were service users involved in the development of the measure?** |
| --- | --- | --- | --- | --- | --- | --- | --- |
| HSE Service User Experience Survey | 2018 | HSE National Addiction Advisory Governance Group 2018^25^ | access, dignity and respect, safe and effective services, communication and information, participation, privacy, improving health, accountability | Addiction Treatment Services: all program types | Eight Community Health Organisation areas Ireland | Not assessed/ reported | Yes |
| Ontario Perception of Care Tool for Mental Health and Addictions (OPOC-MHA) | 2008 | Rush et al., 2013^26^ | Access/entry; services provided; participation/rights; therapists/support workers/staff; environment; discharge/finishing the program/treatment; overall experience | Residential, inpatient, day programs, out-patient programs and supports, community based counselling, psychosocial support, detoxification (other mental health settings were also in scope) | Pilot of 83 programs in single state-level jurisdiction; designed for use across the jurisdiction; Canada | Factor analysis (EFA, factor structure, CFA), internal consistency, concurrent validity | Yes |
| Patient Experience Survey | Around 2018 | Liu, Currie & Adamyk-Simpson, 2018^27^; Currie et al., 2020^28^ | access, humanity of care, skill and quality of staff, engagement, intra and extra-program communication, treatment, continuity of care | AOD and mental health inpatient and outpatient programs | Programs across jurisdiction (Alberta Health Services, Canada) | face validity, factor (EFA), convergent validity, predictive validity, test-retest, internal consistency | Yes |
| Patient Experiences Questionnaire for Interdisciplinary Treatment for Substance Dependence (PEQ-ITSD) | Around 2013 | Haugum et al., 2017^29^ | reception and waiting time; the therapists/the personnel; the treatment; the milieu and activity provision; preparations for the time after discharge; other assessments; previous admissions in substance dependence institutions | Public and private residential services (excluding detoxification) | National survey; Norway | Factor (EFA), internal consistency, test-retest reliability, construct validity | Yes |
| Patient-Reported Experience Measure for Addiction Treatment (PREMAT) | Around 2019 | Hinsley, Kelly & Davis, 2019^30^; Kelly et al., 2021^31^ | Access to care; respect for preferences, values and expressed needs, coordination of care, information and communication, physical comfort, emotional support, involvement of family and friends, continuity and transition | Residential care, including 3 therapeutic communities and a cognitive behavioural therapy based residential program; 12-week day program | 5 services in single state-level jurisdiction; Australia | Face validity, factor structure (PCA), internal consistency, test-retest reliability, convergent and divergent validity | Yes |
| South African Addiction Treatment Services Assessment (SAATSA), and an adaptation for adolescents | 2008 – 2013 | Myers et al., 2015^32^; Myers et al., 2019^33^ | effectiveness, efficiency, access to treatment, person-centred services, quality of services | Residential, outpatient; including adolescent specific | Tested in multiple services in 2 state-level jurisdictions; designed for national use; South Africa; Tested in 5 adolescent services in 2 state-level jurisdictions; South Africa | face validity, factor analysis (EFA, CFA), construct validity, internal consistency | Yes (adolescent version – original version did not) |
| Un-named measure developed for the ‘Downtown Eastside Second Generation Strategy’ (DTES-2GS) | 2016 | Olding et al., 2018^51^ | Not reported, but based on the WHO responsiveness survey | Multiple health and AOD settings (strategy to provide integrated and coordinated care for people who use drugs) | 3 community organisations in one city; Canada | Not assessed/ reported | Yes |

**References**

1. Strobbe S, Brower KJ and Galen LW. Patient satisfaction with outpatient detoxification from alcohol. *Journal of Addictions Nursing* 2004; 15: 23-29.

2. Madden A, Lea T, Bath N, et al. Satisfaction guaranteed? What clients on methadone and buprenorphine think about their treatment. *Drug and Alcohol Review* 2008; 27(6): 671-678.

3. Stöver H. Barriers to opioid substitution treatment access, entry and retention: a survey of opioid users, patients in treatment, and treating and non-treating physicians. *European Addiction Research* 2011; 17: 44-54.

4. Fischer G and Stover H. Assessing the current state of opioid-dependence treatment across Europe: Methodology of the European quality audit of opioid treatment (EQUATOR) project. *Heroin Addiction and Related Clinical Problems* 2012; 14(3): 5-70.

5. Eurasian Harm Reduction Association. *Survey of client satisfaction with opioid maintenance therapy (OMT) services among patients of OMT programmes in Kyiv and the Kyiv Oblast region. Pilot study report*. 2020. Vilnius, Lithuania: EHRA.

6. Kirouac M, Witkiewitz K and Donovan DM. Client Evaluation of Treatment for Alcohol Use Disorder in COMBINE. *Journal of Substance Abuse Treatment* 2016; 67: 38-43.

7. Ives M, Funk R, Ihnes P, et al. *GAIN Global Appraisal of Individual Needs Evaluation Manual*. 2012. Illinois: GAIN Coordinating Center, Chestnut Health Systems.

8. Congia P, Tarantini F, Paolo Pani P, et al. Exploring components and correlates of satisfaction in an outpatient drug addiction service. *Italian Journal on Addiction* 2012; 2: 6-12.

9. Best D. *The NTA's 2005 survey of user satisfaction in England*. 2007. London: National Treatment Agencyfor Substance Misuse.

10. Forman R, Crits-Christoph P, Kaynak Ö, et al. A feasibility study of a web-based performance improvement system for substance abuse treatment providers. *Journal of substance abuse treatment* 2007; 33: 363-371.

11. Hogan B, Hershey L and Ritchey S. A case study using a patient satisfaction survey to improve the delivery and effectiveness of drug addiction treatment services: marketing implications and organizational impact. *Health marketing quarterly* 2007; 24(1-2): 93-106.

12. Eisen SV, Wilcox M, Idiculla T, et al. Assessing consumer perceptions of inpatient psychiatric treatment: the perceptions of care survey. *The Joint Commission journal on quality improvement* 2002; 28(9): 510-526.

13. Barry DT, Moore BA, Pantalon MV, et al. Patient satisfaction with primary care office-based buprenorphine/naloxone treatment. *J Gen Intern Med* 2007; 22: 242-245.

14. Kehoe P, Wodak A and Degenhardt L. *Patient satisfaction in a NSW public opioid pharmacotherapy clinic: measurement and responses*. National Drug and Alcohol Research Centre, University of New South Wales, 2004.

15. Petrar S, Kerr T, Tyndall MW, et al. Injection drug users' perceptions regarding use of a medically supervised safer injecting facility. *Addictive Behaviors* 2007; 32(5): 1088-1093.

16. Alcohol Tobacco and Other Drug Association ACT (ATODA). *Service Users' Satisfaction and Outcomes Survey 2018: a census of people accessing specialist alcohol and other drug services in the ACT*. 2020. Canberra: ATODA.

17. Howard J and Arcuri A. Predictors of retention, and client perceptions of treatment satisfaction and outcomes, among young people presenting to residential drug and alcohol treatment with alcohol as a primary or secondary substance of concern. *Australia: Ted Noffs Foundation* 2005.

18. Cole TO, Robinson D, Kelley-Freeman A, et al. Patient Satisfaction With Medications for Opioid Use Disorder Treatment via Telemedicine: Brief Literature Review and Development of a New Assessment. *Frontiers in public health* 2021; 8: 557275.

19. Joe GW, Broome KM, Rowan-Szal GA, et al. Measuring patient attributes and engagement in treatment. *Journal of substance abuse treatment* 2002; 22: 183-196.

20. Marsden J, Stewart D, Gossop M, et al. Assessing client satisfaction with treatment for substance use problems and the development of the treatment perceptions questionnaire (TPQ). *Addict Res Theory* 2000; 8: 455-470. Article.

21. Stallvik M, Flemmen G, Salthammer JA, et al. Assessing health service satisfaction among users with substance use disorders within the municipalities in Norway. *Subst Abuse Treat Prev Policy* 2019; 14: 18.

22. Dhumal T. *Patient Satisfaction with Substance Use Disorder Rehabilitation Services*. M.S., Duquesne University, Ann Arbor, 2019.

23. Lea T, Sheridan J and Winstock A. Consumer satisfaction with opioid treatment services at community pharmacies in Australia. *Pharm World Sci* 2008; 30: 940-946.

24. Perez de los Cobos J, Valero S, Haro G, et al. Development and psychometric properties of the Verona Service Satisfaction Scale for methadone-treated opioid-dependent patients (VSSS-MT). *Drug Alcohol Depend* 2002; 68: 209-214.

25. HSE National Addiction Advisory Governance Group. *National Summary of the Service User Experience Survey 2017*. 2018. HSE National Social Inclusion Office.

26. Rush B, Hansson E, Cvetanova Y, et al. *Development of a client perception of care tool for mental health and addictions: Qualitative, quantitative, and psychometric analysis: Final report for the Ministry of Health and Long-Term Care*. Centre for Addiction and Mental Health, 2014.

27. Liu P, Currie S and Adamyk-Simpson J. What are the most important dimensions of quality for addiction and mental health services from the perspective of its users? *Patient Experience Journal* 2018; 5: 106-114.

28. Currie SR, Liu P, Adamyk-Simpson J, et al. Validation of a Comprehensive Patient Experience Survey for Addiction and Mental Health that was Co-designed with Service Users. *Community Mental Health Journal* 2020; 56: 735-743.

29. Haugum M, Iversen HH, Bjertnaes O, et al. Patient experiences questionnaire for interdisciplinary treatment for substance dependence (PEQ-ITSD): Reliability and validity following a national survey in Norway. *BMC Psychiatry* 2017; 17(1) (no pagination).

30. Hinsley K, Kelly PJ and Davis E. Experiences of patient-centred care in alcohol and other drug treatment settings: A qualitative study to inform design of a patient-reported experience measure. *Drug and alcohol review* 2019; 38(6): 664-673.

31. Kelly PJ, Hatton EL, Hinsley K, et al. Preliminary psychometric evaluation of the patient reported experience measure for addiction treatment (PREMAT). *Addict Behav* 2021; 123: 107048.

32. Myers B, Govender R, Koch JR, et al. Development and psychometric validation of a novel patient survey to assess perceived quality of substance abuse treatment in South Africa. *Substance abuse treatment, prevention, and policy* 2015; 10: 44.

33. Myers B, Johnson K, Lucas W, et al. South African service users' perceptions of patient-reported outcome and experience measures for adolescent substance use treatment: A qualitative study. *Drug Alcohol Rev* 2019; 38: 823-830.
