# Supplementary material for: Involvement of people who use alcohol and other drug services in the development of patient‐reported measures of experience: A scoping review
Source: Health Expect. 2023 Jul 29;26(6):2151–63. doi: 10.1111/hex.13829 (PMC10632652; doi:10.1111/hex.13829)
Supplement: Supplementary file 4 — Supporting information. [file HEX-26--s002.docx]

**Supplementary Table 4: Stages and activities of service user involvement in measure development with excerpts of text from each article describing the development of the measure** (shaded rows indicate activities where AOD service users were involved in that stage of measure development; unshaded rows indicate no, ‘not reported’ or inadequately described AOD service user involvement)

**Key: Description of stages and activities**

| **Stage of development** | **Activities describing involvement by AOD service users and other stakeholders in each stage** | |
| --- | --- | --- |
| 1. **Research activities and/or governance processes**   Describes how and who was involved in governance processes such as Advisory Groups, and in the research process (e.g. data collection, analysis) | 1 | AOD service users and/or representatives of peer-based consumer organisations are members of Advisory Group or have formal input mechanism to Group |
|  | 2 | Formal research partnership with peer-based consumer organisations (e.g. memorandum of understanding; partnership agreement; formal collaboration) |
|  | 3 | Peer researchers (i.e. people with lived experience of accessing AOD services) are leading the research, or have formal roles in the research team and are involved in data analysis/ interpretation |
|  | 4 | Peer researchers (i.e. people with lived experience of accessing AOD services) have formal roles in the research team and are involved at least in data collection; and/or assistance in data collection provided by peer-based organisation |
|  | 5 | AOD service users involvement in governance processes and/or research activities (other than as participants) is not reported or adequately described |
| 1. **Determining experience dimensions or themes**   Describes how and who was involved in determining which experience-dimensions or themes will be measured | 1 | Involvement by AOD service users in determining the content of topic guides for focus groups/ interviews |
|  | 2 | AOD service users participate to identify dimensions or themes of experiences of service use (e.g. through interviews, focus groups or Advisory Group mechanisms) |
|  | 3 | AOD service users provide input into interpretation of dimensions or themes and/or naming of domains |
|  | 4 | Input from peer-based consumer groups into dimension or theme development |
|  | 5 | Dimensions/themes of experience are derived from the literature, theory or other experts whose peer status is not confirmed |
|  | 6 | Method for determining experience dimensions/themes is not reported or adequately described |
| 1. **Item development** Describes how and who was involved in developing items corresponding to each of the dimensions or themes | 1 | AOD service users involved in generating items against each theme (e.g. through focus group discussions, interviews or other mechanisms, such as advisory structures) |
|  | 2 | Peer-based consumer groups involved in generating items against each theme |
|  | 3 | Items are developed from literature, theory, or other experts whose peer status is not confirmed |
|  | 4 | Method for item development is not reported or adequately described |
| 1. **Survey refinement and finalisation**   Describes how and who was involved in reviewing and finalising the draft survey | 1 | Review of, and feedback provided, on one or more versions of draft survey(s) by AOD service users and/or peer-based consumer groups (including through Advisory structures) |
|  | 2 | Review of, and feedback provided, on one or more versions of draft survey(s) by stakeholders (other than researchers), such as service providers, or policy makers |
|  | 3 | Only researchers involved in review, or method for survey refinement and finalisation is not reported or adequately described |
| 1. **Testing for comprehensibility and/or acceptability**   Describes how and who was Involved in testing the questionnaire to ensure that it is understandable and interpreted correctly | 1 | Questionnaire tested with formalised process of testing for comprehension and clarity with AOD service users (e.g. cognitive interviews) |
|  | 2 | Questionnaire tested with AOD service users or peer-based consumer groups with opportunities given to provide feedback |
|  | 3 | Questionnaire pilot tested with AOD service users with no formal mechanism for feedback reported |
|  | 4 | Questionnaire pilot tested with other stakeholders (e.g. service providers) with or without formal mechanism for feedback |
|  | 5 | Method for testing for comprehensibility is not reported or adequately described |

| **Patient-reported experience/ satisfaction measure** | **Excerpts from text describing the development of the measure** | **Stages (A – E) and activities (1 – 6) of involvement described  (see reference table above)** | | | | | | | | | | | | | | | | | | | | | | | |
| --- | --- | --- | --- | --- | --- | --- | --- | --- | --- | --- | --- | --- | --- | --- | --- | --- | --- | --- | --- | --- | --- | --- | --- | --- | --- |
|  |  | **A** | | | | | **B** | | | | | | **C** | | | | **D** | | | | **E** | | | | |
|  |  | **1** | **2** | **3** | **4** | **5** | **1** | **2** | **3** | **4** | **5** | **6** | **1** | **2** | **3** | **4** | **1** | **2** | **3** | **1** | | **2** | **3** | **4** | **5** |
| Chelsea Arbor Treatment Center Patient Satisfaction Survey Outpatient Detoxification^1^ | - we developed a Patient Satisfaction Survey (PSS) to assist in the evaluation of the program (p.24)^1^ - the survey instrument used to measure patient satisfaction was created as part of an internal quality improvement project, and was not subject to the rigors of formal psychometric design. (p.28)^1^ |  |  |  |  | 🗸 |  |  |  |  |  | 🗸 |  |  |  | 🗸 |  |  | 🗸 |  | |  |  |  | 🗸 |
| Consumer Satisfaction Survey for people accessing opioid maintenance therapy^2^ | - The aim of the current study was to assess consumer satisfaction with opioid treatment services across a number of domains, including the clinic environment, service provision, clinical relationships, medication and treatment outcomes. This was undertaken by Drug Health Services, Sydney South West Area Health Service in partnership with the Australian Injecting and Illicit Drug Users’ League (AIVL), the peak body representing state and territory drug user organisations and issues of national significance for people who use or have used illicit drugs in Australia. (p.672)^2^ - An interviewer-administered questionnaire was developed in collaboration with treatment providers and drug-user organisations, and was supported by a literature review of relevant research. The questionnaire explored demographics, treatment history, clinical practice, global treatment satisfaction, relations with case managers and prescribers, consumer involvement in treatment planning, satisfaction with key domains of case management and satisfaction with aspects of service delivery at the clinic. Three items from the Treatment Perceptions Questionnaire (TPQ) were included. Open-ended questions invited clients to indicate the best and worst aspects of treatment and the clinic, and what they would like to change about treatment and the clinic. (p.672).^2^ - An attempt was made to allow for these differences by using interviewers from AIVL who had an understanding of barriers and issues for pharmacotherapy consumers. (p. 672)^2^ - One of two representatives from AIVL (NB, AM) attended each clinic for 1 week during weekday dosing hours, where they interviewed clients presenting for supervised dosing. (p.672)^2^ - Responses to open-ended questions were coded by three researchers (AM, NB, TL) (p.672) [AM is from AIVL; see author affiliations]^2^ |  | 🗸 | 🗸 | 🗸 |  |  |  |  | 🗸 | 🗸 |  |  | 🗸 | 🗸 |  |  |  | 🗸 |  | |  |  |  | 🗸 |
| EQUATOR (European Quality and Audit of Opioid Treatment)^3, 4^ | - The questionnaire and interview instruments were piloted and refined prior to implementation of the study (p.46).^3^ - The author wishes to thank … Urs Koethner and Dirk Schäffer for assistance in developing the questionnaires and assisting to implement the study (p.53).^3^ - All authors were involved in survey and questionnaire design….HS [Heino Stover – one of the authors] designed the original Project IMPROVE questionnaires… (p.14).^4^ |  |  |  |  | 🗸 |  |  |  |  |  | 🗸 |  |  |  | 🗸 |  |  | 🗸 |  | |  | 🗸 |  |  |
| Eurasian Harm Reduction Association survey^5^ | - Our approach to this study was based on the principles of equal partnership with the community of people who inject drugs and/or receive opioid maintenance therapy (OMT). This community was represented during this project by partners from four organizations: the Ukrainian Network of People who use Drugs (VOLNA), Drug-users Ukraine, the Drop-in Centre and the ENEY. (p.2)^5^ - It was reasonable to fill that gap by conducting a short qualitative survey to form a set of questions to measure satisfaction with OMT services using data collected in Ukraine. (pp. 7-8)^5^ - As a result, eight focused interviews and eight interviews with respondents from another study were conducted, with respondents being asked about their experience in OMT programmes. For data analysis in our research, we used transcripts of the interviews and field notes….Within the scope of the study, the main purpose of semi-structured interviews was to highlight various aspects and criteria of satisfaction with OMT services among patients in order to develop a data-based questionnaire tool. (p.21)^5^ - It is important to discuss the outcomes of the qualitative component with the community. This will ensure they are actively involved in the development of further updates for the quantitative portion of the study. Community members and future interviewers also can test the questionnaire with their peers during this discussion to see whether the questions are clear and easy-to-understand, and how long it would take to complete the questionnaire. At this stage, it is also advisable to discuss and determine the sampling strategies and to collect data on various OMT sites and methods of dispensing medication.(p.60)^5^ - In our study, we had a team of four interviewers who recruited participants. This team consisted of two female interviewers and two male interviewers from four different organizations working in the field of advocacy and the prevention of HIV and other diseases among people who inject drugs.(p.61)^5^ |  | 🗸 |  | 🗸 |  |  | 🗸 |  |  |  |  |  |  |  | 🗸 |  |  | 🗸 |  | |  |  |  | 🗸 |
| Evaluation of Treatment (EOT) measure^6^ | - To this end, the researchers in COMBINE constructed a self-report questionnaire to systematically assess the client evaluations of AUD treatment. (p.39)^6^ - The EOT followed a theory-driven approach to measure construction, assessing several different theoretically relevant domains, including the therapeutic relationship, environmental factors, and client factors. (p.39)^6^ - the EOT measure was developed by researchers and may, therefore omit aspects of treatment satisfaction that may be more apparent to clients or clinicians than to the researchers themselves. (p.42)^6^ |  |  |  |  | 🗸 |  |  |  |  | 🗸 |  |  |  | 🗸 |  |  |  | 🗸 |  | |  |  |  | 🗸 |
| Global Appraisal of Individual Needs (GAIN) subscales: Treatment Satisfaction Index (TSI)/ Treatment Satisfaction Scale (TxSS)^7^ | - It has been developed over more than a decade of collaboration between researchers, treatment programs, policy makers, and information technology specialists. (p.1)^7^ |  |  |  |  | 🗸 |  |  |  |  | 🗸 |  |  |  | 🗸 |  |  |  | 🗸 |  | |  |  |  | 🗸 |
| Multilevel Satisfaction Evaluation (MSE)^8^ | - By means of anonymous questionnaires patients were asked to express their degree of satisfaction in services provided while in treatment. After explaining the aims of the study and providing instructions on the use of instruments and the confidentiality of information to be collected, interviewers handed out the questionnaires, requesting that they be returned within one week.(p.7)^8^ - Moreover, two specific instruments were applied to assess satisfaction, the first of which was the Treatment Perception Questionnaire (TPQ), already used in similar studies.(pp.7-8)^8^ - The second purpose-constructed instrument, “Multilevel Satisfaction Evaluation” (MSE), was used to collect information pertaining to the following areas (p.8)….^8^ - All questions were specifically aimed at obtaining patient evaluation of aspects of the service program not specifically covered by TPQ.(p.8)^8^ |  |  |  |  | 🗸 |  |  |  |  |  | 🗸 |  |  |  | 🗸 |  |  | 🗸 |  | |  |  |  | 🗸 |
| National Treatment Agency for Substance Misuse survey of user satisfaction (NTA)^9^ | - piloted initially as an interview at a community drug service in Birmingham. Following the initial pilot, a self-completion version was developed to address both service delivery factors and attitudes towards a range of aspects of treatment provision. The questionnaire itself was constructed from a number of existing instruments, amended to suit the UK context and the substance misuse nature of the population. (p.5)^9^ - staff piloted the survey across 11 services in the Barking and Dagenham area of London, and Gateshead in the North East of England (six and five services respectively) and with two user groups (p.5)^9^ - The feedback received from the main pilot was that the questionnaires were reasonably easy to complete for clients although time consuming, but that the main issue was around ensuring that clients had sufficient opportunity to receive a questionnaire and the support necessary to complete and return it. For a national survey, it was not possible to use an interviewer led approach and it was essential that treatment services supported the project by ensuing distribution, encouraging completion and assisting with collation and return of the questionnaires. As a consequence, all the drug treatment services identified as providing structured Tier 3 or Tier 4 services were asked to host materials, promote the study, assist with the questionnaire completion and co-ordinate the return of completed questionnaires. An additional sample of needle exchange services were also asked to assist with questionnaire distribution. Clients receiving treatment at more than one service were encouraged to complete a questionnaire for each service they attended. The service was treated as the unit of analysis.^9^ |  |  |  |  | 🗸 |  |  |  |  | 🗸 |  |  |  | 🗸 |  |  |  | 🗸 |  | |  | 🗸 |  |  |
| Patient Feedback Survey^10^ | - The PF survey was developed in accordance with quality improvement indicator development guidelines and with input from 22 clinic supervisors and 18 researchers. Beginning with a pool of potential items, PF survey development team members ultimately selected 12 items for inclusion in the PF survey. Selected items met two quality improvement criteria: (1) the item monitored something that the staff could improve and (2) improvement in the item’s domain was thought to be associated with increases in outcomes that are important to the organization. Three survey item domains were ultimately agreed upon: (1) therapeutic alliance, (2) group treatment satisfaction, and (3) self-reported substance use. The survey also included a domain for demographic information. (p.365)^10^ - The therapeutic alliance scale used in PF was a 4-item scale derived from items from the widely used 24-item California Psychotherapy Alliance Scale. (p.365)^10^ - The treatment satisfaction items were adapted from the Experience of Care and Health Outcomes (ECHO) survey, (p.365)^10^ |  |  |  |  | 🗸 |  |  |  |  | 🗸 |  |  |  | 🗸 |  |  | 🗸 |  |  | |  |  |  | 🗸 |
| Patient Satisfaction Survey^11^ | - The study was developed collaboratively between the methadone clinic management staff and faculty from a local medical college. (p.99)^11^ - The survey instrument was developed to reflect the indexes for patient satisfaction that have been established in the literature including: access to care, general satisfaction, provider conduct, outcome of care, financial aspects of care, availability of care, and continuity of care. After consultation with the managers and counselors, the survey instrument was personalized to the organization through the addition of items of particular interest to the organization. For instance, as a result of a patient complaint, management wanted to know whether or not patients felt that there was racial discrimination toward patients by staff. Similarly, much effort had been given to train counselors toward consistency in clinical approaches, yet there was no information about patient satisfaction with their counselor or whether or not patient satisfaction varied between counselors. Since the counselor-patient relationship is integral to successful treatment, these measures could be of substantial value impact to the organizational strategy. Similarly, the way clients are treated by support staff had reportedly been problematic, so additional items were added that would provide related information. Other items of importance to mental health services and methadone treatment in particular were added. The final instrument included 85 Likert scale items, 14 additional closed-ended response items, and six open-ended questions. (pp.99 – 100).^11^ |  |  |  |  | 🗸 |  |  |  |  | 🗸 |  |  |  | 🗸 |  |  |  | 🗸 |  | |  |  |  | 🗸 |
| Perceptions of Care Survey^12^ | - We [S.V.E., B.D.] began survey development with a review of existing measures of consumer assessments of inpatient and outpatient medical and psychiatric care. From these measures we extracted items pertaining to the interpersonal aspects of care, and we adapted them when appropriate to psychiatric treatment programs. In addition, on the basis of earlier work, we eliminated redundant items and added items unique to inpatient mental health care. (p.511)^12^ |  |  |  |  | 🗸 |  |  |  |  | 🗸 |  |  |  | 🗸 |  |  |  | 🗸 |  | |  |  |  | 🗸 |
| Primary Care Buprenorphine Satisfaction Scale (PCBSS)^13^ | - We developed a patient satisfaction measure that assessed domains explicated by patients in our previous clinical trials and by Novick et al. (p.243) [on following these up with the citations provided, ‘domains explicated by patients’ was based on clinician observations and as determined by practitioners, not on client/patient input]^13^ |  |  |  |  | 🗸 |  |  |  |  | 🗸 |  |  |  |  | 🗸 |  |  | 🗸 |  | |  |  |  | 🗸 |
| Rankin Court measure^14^ | - The project’s work was overseen by a reference group comprising representation from stakeholders including:   - NSW Department of Health   - St Vincent’s Hospital Alcohol and Drug Services   - St Vincent’s Hospital Quality Improvement Unit   - Methadone Advice and Complaints Service   - NSW Users and AIDS Association (NUAA)   Input to the work of the Reference Group was also sought from the body of patients at Rankin Court. (p.3)^14^   - The expertise of senior clinicians in the field and other stakeholders in academia was also drawn on at key milestones throughout the project’s development, through an electronic consultation forum. (p.3)^14^ - Conceptual dimensions underpinning the eleven items referenced those of the original Verona Service Satisfaction Scale (VSSS-32) and the Client Satisfaction Questionnaire (CSQ-8) (p.16)^14^ - For this research, a project officer had undertaken semi-structured interviews with thirty nine patients from three inner city Sydney pharmacotherapy clinics. Thematic analysis of the data identified similar conceptual dimensions to those from the above literature as being the important aspects of service on which to measure patient satisfaction with service in pharmacotherapy clinics for opioid dependent persons, with the inclusion of one additional theme: patients’ input to decision making in health care. (p.16)^14^ - Face validity of the questionnaire was addressed through consultation with senior clinicians working in the field of pharmacotherapy for opioid dependent patients, and by two rounds of pre-testing with a total of twenty subjects from inner urban Sydney pharmacotherapy clinics. Subjects who completed the draft questionnaire for this purpose were interviewed following the pre test, and asked if the instrument excited any discomfort, if any questions were difficult to understand, and if any questions constrained them from saying more than they would have wished. Subjects were also asked to rephrase the questions in their own words, with their responses coded as fully correct, generally correct (no more than one part altered or omitted), partially wrong (general subject still correct), or completely wrong/ no response. Specific terms that had been referred to with multiple meanings in the qualitative data and were used in the questionnaire (dose, treatment, services), were checked for repeatability during the pretest. One of the twenty subjects required the questionnaire to be read to him due to illiteracy. Nine items from across all of the conceptual dimensions were discarded from the questionnaire as a result of this pretesting, due both to the requirements for brevity and to the pre-test subjects’ comments. (p.17)^14^ - Patients who consented to participate were directed to a representative of the drug users advocacy organisation, the NSW Users and AIDS Association (NUAA), for assistance in reading and responding to the questionnaire should such assistance be required (p.18)^14^ | 🗸 | 🗸 |  | 🗸 |  |  | 🗸 |  |  | 🗸 |  |  |  | 🗸 |  |  |  | 🗸 | 🗸 | |  |  | 🗸 |  |
| Scientific Evaluation of Supervised Injecting (SEOSI) questionnaire^15^ | - The items chosen for evaluation in the present study were based upon pilot interviews with injection drug users in the neighborhood prior to the SIF's opening (p.1089)^15^ |  |  |  |  | 🗸 |  | 🗸 |  |  |  |  |  |  |  | 🗸 |  |  | 🗸 |  | |  |  |  | 🗸 |
| Service Users’ Satisfaction and Outcomes Survey (SUSOS)/ Service Users’ Satisfaction Survey (SUSS)^16^ | - ATODA acknowledges the support and input from the ACT specialist Alcohol and Other Drug (AOD) services Executive Directors’ Group and the ACT Alcohol Tobacco and Other Drug (ATOD) Workers’ Group for the 2018, and previous, surveys. The 2018 survey was piloted by the ACT ATOD Workers’ Group, and we thank them for their significant input into the design of the 2018 Survey. (p.iii)^16^ - ATODA also acknowledges the significant contribution of the ACT peer-based consumer group (CAHMA) at each wave of the Survey, including managing the data collection in 2009. At each subsequent wave, consumer engagement (through CAHMA) has been central to receiving input into the questionnaire content (including the inclusion of new questions about consumer participation), and advice on the appropriate implementation of the Surveys (including setting reimbursement amounts). (pp. iii-iv)^16^ - As with previous SUSS and SUSOS surveys, the 2018 Survey includes a validated instrument called the Client Satisfaction Questionnaire—8 (CSQ-8)….The 2018 SUSOS was also informed by the *Queensland Alcohol and Other Drug Treatment & Harm Reduction Outcomes Framework* which was used to identify key outcomes indicators that could be included in the survey. We sought to use relevant questions that had been part of previous Service User Satisfaction Surveys (2009 and 2012) and the 2015 SUSOS, and that had come from the instrument used by the United Kingdom National Treatment Agency for Substance Misuse (pp.3-4).^16^ - This 2018 version of the SUSOS questionnaire received further input from Executive Directors of ACT specialist AOD services, from the ACT ATOD Workers Group, and from the Canberra Alliance for Harm Minimisation and Advocacy (CAHMA) [peer consumer advocacy group]. These inputs informed the modification or inclusion of several survey items, in particular demographic items (e.g. gender identity and sexual orientation), and the wording of questions (p.4).^16^ |  | 🗸 |  |  |  |  |  |  | 🗸 | 🗸 |  |  | 🗸 | 🗸 |  |  | 🗸 |  |  | |  |  | 🗸 |  |
| Ted Noffs Foundation Substance Use Assessment Part C^17^ | - To achieve this, the TNFYSUAA both includes original items and incorporates a number of existing assessment tools. (p.5)^17^ - The TNFYSUAC was designed to measure client perceptions of PALM in the areas of ……. (p.5)^17^ |  |  |  |  | 🗸 |  |  |  |  |  | 🗸 |  |  | 🗸 |  |  |  | 🗸 |  | |  |  |  | 🗸 |
| Telemedicine-delivered medications for opioid use disorder (t-MOUD)^18^ | - we report on the development and proof of concept implementation of a novel patient satisfaction survey specific to tMOUD. We based our survey on characteristics of previously published patient satisfaction surveys, utilizing the following thematic categories to frame the patient’s subjective experience: (i) communication; (ii) privacy; (iii) patient perceptions; and (iv) technology utilization. These categories represent areas on which to assess general satisfaction, and have been implemented in other arenas of research, including the delivery of general mental health services, telemedicine for alcohol use disorder, and telemedicine consultations for mental health disorders. In addition to those four thematic categories, our survey also includes a thematic category to capture (v) treatment access, pertinent to understanding patient satisfaction with the availability of care. (p.3)^18^ - Using thematic categories interpreted from previous published literature, we created a survey that assessed satisfaction with: (p.3)^18^ - AB developed the patient satisfaction survey. (p.6)^18^ |  |  |  |  | 🗸 |  |  |  |  | 🗸 |  |  |  | 🗸 |  |  |  | 🗸 |  | |  |  |  | 🗸 |
| Texas Christian University – Client Evaluation Form (CEF)/Treatment Engagement Form (TEF) from Client Evaluation of Self and Treatment (CEST)^19^ | - The CEST is a self-report instrument with demonstrated utility. It was developed, administered, and researched over a 10-year period in conjunction with grants to develop drug abuse treatment strategies to help reduce dropout, relapse rates, and AIDS-risky behaviors among injecting drug users (p.184)^19^ - That early work and subsequent refinements have culminated in an intake assessment (the ‘‘Client Evaluation of Self at Intake’’ or CESI) and a parallel version for during treatment assessment (the CEST). (p.184)^19^ - the present article is needed to update this information and present new reliability and validity assessments for our latest revisions based on a large, multiprogram sample. (p.185)^19^ - Over the past decade, our research into treatment process issues has focused on measuring elements of treatment engagement, including counseling session attendance and interactions between patient and counselor. Within the latter area, our focus has been on the therapeutic relationship, examining it from both the patient and counselor perspectives. From patient evaluations, measures of counselor respect, treatment satisfaction, and treatment environment were developed, and these proved useful in during-treatment evaluations and in the modeling of time in treatment and posttreatment outcomes. This work has culminated in a generic, cross-modality assessment of treatment process in the CEST that includes sections focused on treatment needs. (p.187)^19^ |  |  |  |  | 🗸 |  |  |  |  | 🗸 |  |  |  | 🗸 |  |  |  | 🗸 |  | |  |  |  | 🗸 |
| Treatment Perceptions Questionnaire (TPQ)^20^ | - We first established an initial pool of 14 items for the TPQ from a review of existing instruments and research literature, and from semi-structured interviews with eight subjects (four in in-patient treatment and four attending community treatment). During the interview, each client was invited to talk freely about their impressions of the service and their treatment and what they found satisfying and dissatisfying about it. A content analysis of the client interviews indicated that two broad areas were central influences on the extent of satisfaction with the treatment received. The first area concerned the perception of clients towards the nature and extent of contact with the programme staff. The second area concerned aspects of the treatment service and its operation and rules and regulations. We compiled the comments from clients and translated these into fourteen belief statements, using both positively and negatively worded items to minimise the likelihood of response set (pp.458-459)^20^ - These items were included in the field-tested version of the TPQ (p.459)^20^ - These items differ substantially from - the CSQ-8 - arguably the most widely used existing client treatment satisfaction measure. They also focus on distinct issues of importance to the study population. (p.459)^20^ |  |  |  |  | 🗸 |  | 🗸 |  |  | 🗸 |  |  |  | 🗸 |  |  |  | 🗸 |  | |  | 🗸 |  |  |
| Un-named 19-item satisfaction survey (Norwegian Municipalities)^21^ | - A partial explorative survey based study specifically designed in collaboration with users, the Norwegian Public Health Institute and researchers at the center of regional drug and alcohol competence center research department in the mid-region of Norway. (p.3)^21^ - The users were involved in the design of the study by being a reference group for the project throughout the entire project period. Here they could bring in relevant questions to be included in the survey regarding satisfaction and other issues they had with the study. The survey was then tested among users in the relevant target group who filled it out and reported back to the principal investigator. A focus group with 4 users was established with the project group and the questionnaire items were discussed with the users resulting in minor revisions to the final questionnaire to improve face validity. (p.3)^21^ - Items covering satisfaction were specifically designed to investigate what kind of services they had used, to what degree the services helped them in important life areas, whether they had received practical help and experiences with the personnel and overall service satisfaction. The 19 Satisfaction items were graded on a five-point Likert scale from “not at all” to “a large extent”. (p.4)^21^ - The need for a broad approach when investigating what is associated with satisfaction among users of SUD services within the municipalities warrants the questions in our survey. Some of them are taken from satisfaction surveys previously executed on inpatient patients in specialized service, but some were added based on focus group executed together with the users who stated their importance to measure their satisfaction. (p.9)^21^ | 🗸 |  |  |  |  |  | 🗸 |  |  | 🗸 |  | 🗸 |  | 🗸 |  | 🗸 |  |  |  | | 🗸 |  |  |  |
| Un-named patient satisfaction survey (residential rehabilitation)^22^ | - The second phase of the study consisted of development and pilot testing of a standardized patient satisfaction questionnaire. The results of the qualitative analysis were applied in the conception of items for the questionnaire tool. The questionnaire was then assessed for face validity and suggestions elicited from the clinical staff were incorporated in the questionnaire. The tool was pilot-tested in a sample of 17 patients seeking treatment for SUD at the facility for at least two weeks. (p.v)^22^ - Phase I of the study utilized a semi-structured interview approach with open-ended questions to explore the characteristics of patient satisfaction relevant to the treatment program at Salvation Army Harbor Light Center. An extensive literature review formed the basis for the semi-structured interviews conducted individually with the participants. Directed content analysis of the interviews was implemented to identify the dimensions relevant to patient satisfaction. (p.56)^22^ - Preliminary literature search (and initial assessment of articles before the exhaustive systematic literature review) aided in identification of critical aspects related to patient satisfaction. This evaluation determined the following areas for exploration: (1) facility expectations, (2) overall programmatic structure, (3) effectiveness of counseling case session, (4) counselor expertise, and (5) effectiveness of referral services. These potential areas were utilized in the development of open-ended questions for the semi-structured interviews. Numerous discussions with the research committee focused within each major area to gather additional information related to several aspects underlying the major domains of interest. Sub-domains were identified, and related prompts were created, for example, within facility expectations, prompts were created focusing upon information regarding adequacy of physical space, privacy, and safety-security. All the domains and sub-domains were selected on the basis of their potential of being associated with patient’s satisfaction with the treatment. (p.59)^22^ - The interviewer (TD [student researcher]) was thoroughly trained by a faculty committee member (VG), skilled in qualitative methods with an expertise in SUD and mental illness, before executing the interviews (p.61)^22^ - The study included multiple coders to analyze the interview data; thus, it was important to establish consistency in coding between these coders. Before starting the coding process, the researcher provided the two junior graduate students with an overview of the study objectives and process. This research team was informed about their roles in transcription and coding and were thoroughly instructed with a comprehensive explanation for each step in the process. (pp.64-65)^22^ - Phase II of the study consisted of developing and pilot testing a standardized questionnaire tool assessing patient satisfaction. The qualitative content analysis of the interview data collected in Phase I of the study identified relevant themes and relationships across those themes. The results of the qualitative analysis were utilized in the conception of items for the questionnaire tool. The questionnaire was then assessed for face validity and pilot tested in a sample of patients seeking treatment for SUD. Data obtained from the survey piloting was utilized to assess item reliability, to create scales, and to assess correlations across items. (p.67)^22^ - The research team [including the researcher (TD) and the committee] initiated the process of item development utilizing results obtained from the content analysis of the qualitative interviews as a premise.   The process broadly started with the consideration of major themes from the content and axial analyses. The most frequent theme (e.g. counselor skill) was identified from the frequency distribution of data, and all the relations appropriate to this particular theme was determined from the axial analysis. This process was repeated for the five most frequent themes: counselor skill, programmatic structure (adhering), skill development (personal responsibility), comparison to other programs, and case management facilitation. The identified themes and relations were utilized in the conception and development of each item. The themes ‘counselor skill’, ‘programmatic structure’, and ‘skill development’ served to be major sections of the survey, utilized as individual scales with specific items encompassing it. The items for the theme ‘case management facilitation’ was accommodated under ‘counselor skill’ section of the survey due to overlapping of certain item-related patterns whereas the theme ‘comparison to other programs’ was incorporated into a ‘preference scale’, the fourth specific scale section of the survey. This specific scale being an extension of the theme ‘comparison to other program’ was not primarily focused to assess satisfaction, it was mainly utilized to understand the general preferences of the patients who have attended such treatment programs in the past.  In addition to the items ascertained from the interviews, the questionnaire items also focused on the results obtained from the systematic literature review and thus, each question was designed considering all the necessary aspects. (pp.68-69)^22^   - Face validity was tested to assess whether the questionnaire tool qualified as a subjective representation of what it purports to measure. This was performed by an expert panel inclusive of four counselors and the program director from the Harbor Light facility. The experts were given a feedback form consisting of four questions to receive necessary suggestions and to understand their overall impression of the tool. (p.72)^22^ - Pilot test of the questionnaire tool utilized a cross-sectional descriptive survey methodology. …. After the informed consent was obtained, each participant was handed the questionnaire. Any questions or queries while filling the questionnaire was addressed and resolved by the researcher. After completion of the survey, the researcher thanked the participants and processed reimbursement for their participation. (p.73)^22^ - The item conception step was supervised by researchers trained in survey/questionnaire development and was executed with consideration of minimizing item-related issues and potential overlaps. (p.104)^22^ |  |  |  |  | 🗸 |  | 🗸 |  |  | 🗸 |  |  |  | 🗸 |  |  | 🗸 |  |  | |  |  | 🗸 |  |
| Un-named satisfaction survey for opioid treatment at community pharmacies^23^ | - The questionnaire was developed by the researchers in collaboration with the Australian Injecting and Illicit Drug Users’ League (AIVL), the peak body representing drug user organisations in Australia, as well as senior clinicians and community pharmacists. The questionnaire was piloted with nine clients who provided valuable feedback to improve content and ease of understanding. (p.941)^23^ |  | 🗸 |  |  |  |  |  |  |  | 🗸 |  |  | 🗸 |  |  |  |  | 🗸 |  | | 🗸 |  |  |  |
| Verona Service Satisfaction Scale for methadone-treatment (VSSS-MT)^24^ | - We chose the 32-item Verona Service Satisfaction Scale (VSSS-32), the shortest version of the VSSS, as a starting point for creating an instrument able to specifically assess methadone-maintained patient satisfaction. (p.209)^24^ - We adapted the VSSS-32 to assess opioid-dependent patient satisfaction with services received from methadone treatment centres. (p.210)^24^ - The text of the VSSS-32 was modified, adapting it specifically for methadone maintenance treatment (process not presented). (p.210)^24^ - Four research assistants, three psychologists and one physician were trained during 1 week on how to administer the survey. (p.211)^24^ - Exploratory factor analysis of the preliminary version of the VSSS-MT was conducted, using principal component analysis. (p.211)^24^ - As a result of these refinements, the final version of the scale includes only 27 items. (p.212)^24^ |  |  |  |  | 🗸 |  |  |  |  | 🗸 |  |  |  | 🗸 |  |  |  | 🗸 |  | |  |  |  | 🗸 |
| **Patient-reported experience measures** |  |  |  |  |  |  |  |  |  |  |  |  |  |  |  |  |  |  |  |  | |  |  |  |  |
| HSE Service User Experience Survey^25^ | - The literature review (appendix 3) assisted in the questionnaire development and an audit was conducted of the types of tools currently being used across Addiction Services in Ireland. These were largely specifically developed questionnaires for identifiable service outcomes…..   The key domains of Service User experience are outlined in the literature review and a review was conducted of the most frequently used frameworks applied in Service User experience surveys….  In Ireland, the National Healthcare Charter and the NSSBHC Theme 1, (Appendix 4), condensed in Table 1, could easily be mapped into any of these most commonly used frameworks. There are similarities and overlap between these frameworks and quality standards. (p.8)^25^   - The overall objective was then to establish a limited set of questions, covering the core components of Service User experience. Therefore the questions would be mapped on the National Healthcare Charter and on Theme 1 of the NSSBHC. (p.10)^25^ - The process of designing the Service User experience tool comprised of the following key steps (pp.10-11):^25^   - Agreeing key domains of Service User experience as per Service User charter and Theme 1 NSSBHC   - Design questionnaire : Mapping key questions to each domain in Theme1   - Question compilation from examining research and collaborating with Service User representatives   - Feedback from Advisory Group (NAAGG) [Membership of NAAGG includes HSE Operational Managers responsible for the provision of addiction services throughout the country, Social Inclusion Specialists and the National Rehabilitation Coordinator (p.7)] - Questionnaires were identified from all major Service User experience surveys…. Particular attention was given to the Service User experience surveys used at a national level in the NHS in the UK as these surveys are developed by organisations expert in this area such as the Picker Institute. A list of questions were compiled for each of the nine domains, and further reduced to four questions per domain….   Complaints and response to complaints and concerns is theme 1.8 of the NSSBHC. However, it was not a question in any of the major current survey tools and is not in the frameworks noted in Table 1 above. It was decided that this was an important domain to measure and one additional question was therefore included for complaints. (p.11)^25^   - the questionnaire was pre-tested via mixed qualitative and quantitative measures. …. The pre-pilot phase also demonstrated question comprehension and highlighted changes that need to be made accordingly. The steps taken prior to going to the pilot phase included: self-administrating the Question Appraisal System (QAS 99), obtaining Service User representative feedback, and consulting with the HSE National Quality Improvement & Safety Division. - CHO 1 self-administered the QAS 99, this questionnaire appraisal system was designed by Willis and Lessler (1999) to assist questionnaire designers in evaluating survey questions and in finding and fixing problems before the questions go to pilot or use (p.12)^25^ - Prior to full implementation the tool was further tested through a pilot…..   The survey was tested with the people attending services, with the assistance of UISCE, an organisation representing people who use drugs (p.12)^25^   - Following the pilot, the interviewers and key stakeholders met to review the findings of the pilot under the following broad themes   1) Was it easy to get the Service User to engage with the process?  2) Was there any obvious difficulty with question interpretation?  3) Was the promotional material adequate?  4) Did you as staff feel comfortable introducing the questionnaire?  5) What concerns would you have about Service Users completing the survey?  6) Did you notice any changes in your own behaviour over the two days whilst the pilot was on?  7) Were the questions easily understood?  8) What other questions should be included?  9) Other observations  Amendments to the questionnaire were made following the pilot and prior to full implementation.(p.13)^25^ |  |  |  | 🗸 |  |  |  |  |  | 🗸 |  | 🗸 |  | 🗸 |  | 🗸 |  |  |  | |  | 🗸 | 🗸 |  |
| Ontario Perception of Care Tool for Mental Health and Addictions (OPOC-MHA)^26^ | - Overall, the qualitative feedback indicated that the OPOC-MHA was generally well-accepted and the majority of staff at each pilot site felt that the tool appropriately captured the importance of questions using a perception of care approach. - A broad range of stakeholders were engaged throughout the development processes via a Program Advisory Committee and Working Group (see Appendix B for a list of members). Partners included the Ministry of Health and Long-Term Care, specialized mental health and substance use agencies (directors, managers, clinicians and researchers), Local Health Integration Networks (LHINs), and consumer representatives. Stakeholders informed the decision-making regarding the development of the tool, selection of pilot sites, and procedures for implementation. The OPOC-MHA was reviewed by a separate youth committee consisting of stakeholders working with young people. They offered guidance to ensure the appropriateness of the tool for a younger population. Francophone programs and colleagues supported the translation process. We also received very helpful feedback from the Persons with Lived Experience and Family Member Advisory Panel; for example, they noted that many of the questions, being framed as they are from a perception of care perspective, implies the existence of a standard or standards against which people can reflect on their experience. They challenged whether such standards currently exist, or if they do, if people would be aware of them.(pp.14-15)^26^ - Following the literature review and environmental scan, the tool underwent a rigorous development process. Measures that had been identified in the literature review were examined to determine which tools appeared to be most useful, had the strongest validity and reliability data, and had been used in both mental health and addiction settings. Seven tools met these criteria: Client Satisfaction Questionnaire (CSQ), Service Satisfaction Scale (SSS), Global Appraisal of Individual Needs (GAIN), Experience of Care and Health Outcomes Survey (ECHO), Inpatient Evaluation of Service Questionnaire (IESQ), Mental Health Statistics Improvement Program (MHSIP), and the Verona Service Satisfaction Scale (VSSS). A tool under development at the time by the Centre for Addiction and Mental Health (CAMH) on behalf of Accreditation Canada was also reviewed.(p.15)^26^ - The project team and sub-group of the larger Advisory Committee reviewed each item from all of these tools for its clarity and brevity, conceptual and statistical redundancy with other items, factor loadings on particular sub-scales, and relationship to key agency characteristics and operational processes that could be the target for concrete quality improvement initiatives. Through an iterative process conducted by email, teleconferences and face-to-face meetings, a comprehensive list of items was formed and clustered into eight domains. These items became the foundation for the development of the OPOC-MHA …. (pp.14-15)^26^ - Twenty-three pilot agencies, comprising 83 separate programs, participated in this study (see Appendix D for descriptions of the pilot sites). The sites, listed below in Table 2, represented a cross-section of addiction and mental health agencies in Ontario, and thus included a diversity of programs and clientele (i.e. youth, gender-specific, ethno-cultural, immigrant). (p.17)^26^ - In October of 2012, the research team met with the project Working Group to discuss the pilot site results and feedback. (p.36)^26^ - The wording of a few other items was also revised for clarity and/or cultural sensitivity based on our pilot site feedback and with the support of the Working Group. (p.38)^26^ | 🗸 |  |  |  |  |  | 🗸 |  |  | 🗸 |  | 🗸 | 🗸 | 🗸 |  | 🗸 | 🗸 |  |  | |  | 🗸 |  |  |
| Patient Experience Survey^27, 28^ | - Focus groups were the primary avenue to obtain patient input on the essential dimensions of a high-quality service. In the spirit of the Patient and Family- Centred Care (PFCC) model, we recruited A&MH patients to oversee the project. (p.107)^27^ - In the present project, patients were established early in project development as key decision makers in a manner consistent with the *involving*, *collaborating*, and *empowering* components of the IAP2 model. To this end, a patient and family advisory committee was assembled and had the responsibility to provide consumer input into all aspects of the research process and serve as the primary decision-making body for the project. (p.107)^27^ - The patient and family advisory committee was made up of an experienced group of individuals with lived experience in using A&MH services along with frontline service providers and researchers. At least half of the advisory members were made up of patient and family advisors to ensure that patients were well represented and had a strong voice.(p.107)^27^ - The patient advisors were consulted on formulating the interview guide for the focus groups and assisted in analyzing the anonymous data (p.107)^27^ - The focus groups were conducted in two rounds. The first round used open-ended questions to identify service dimensions that contribute to patients having a positive or negative experience in using the service. (p.107)^27^ - A second round of focus groups was conducted to construct and validate items to assess patient experience within each of the themes. Based on feedback from participants, items were refined to ensure the content captured the experience of A&MH service users.(p.107)^27^ - A small working group composed of patient advisors and researchers was formed to complete the qualitative analysis. Patient advisors and researchers collaboratively generated the coding framework. Two coders were assigned to each focus group transcript. A patient advisor was the first coder and a research team member acted as the second coder…. The final themes were presented and reviewed by the larger advisory committee. (p.108)^27^ - The most innovative aspect of the project was the role of patients in the analysis and reporting process. Patient advisors were an integral part of identifying themes of service experience from focus group transcripts. (p.112)^27^ - Items for the patient experience instrument were constructed based on input from A&MH service users and examining the content of other existing validated patient experience surveys for Addiction and Mental Health or general medical services. Items were generated to cover the seven domains of service identified through the qualitative study. (p.737)^28^ - Hence, the initial draft questionnaire underwent testing with a small sample of patients (n = 18) using cognitive interviewing, a survey development method that builds a comprehensive understanding of how respondents interpret each question. Using procedures such as thinking out loud, cognitive interviews help to refine item wording to ensure consistent comprehension, recall, and decision making for the response options across respondents. (p.737)^28^ - Scale naming was based on the theme suggested by the items, and the input from the patient and family advisory committee. (p.738)^28^ | 🗸 |  | 🗸 |  |  | 🗸 | 🗸 | 🗸 |  |  |  | 🗸 |  | 🗸 |  |  |  | 🗸 | 🗸 | |  |  |  |  |
| Patient Experiences Questionnaire for Interdisciplinary Treatment for Substance Dependence (PEQ-ITSD)^29^ | - The development of the questionnaire followed the standard methodology of our national program, including a literature review, cognitive interviews with patients and expert consultations. (p.2)^29^ - Firstly, a comprehensive literature review was conducted to search for valid and reliable questionnaires that could be used in the Norwegian context…. Questionnaires, both from the review and Norwegian questionnaires that had been used locally, were considered in terms of identifying important and relevant topics for the new questionnaire. (p.3)^29^ - Secondly, an expert group were consulted several times to discuss the content of the new questionnaire, as well as procedures for data collection. The expert group consisted of seven persons, including clinicians/therapists, researchers associated with treatment institutions and representatives from interest groups. (p.3)^29^ - Thirdly, qualitative interviews were conducted with 13 patients with various types of substance dependencies, with a focus on what they found to be important while in treatment. (p.3)^29^ - Fourthly, the resulting questionnaire was cognitively tested with patients (n = 15), and lastly, a pilot survey was conducted with 14 institutions (n = 329). The first version of the questionnaire included 45 questions. (p.3)^29^ - Before the national survey, the questionnaire was expanded with three modified items from the Patient Enablement Instrument, and three questions about help from the municipality. (p.3)^29^ |  |  |  |  | 🗸 |  | 🗸 |  |  | 🗸 |  |  |  | 🗸 |  |  |  | 🗸 | 🗸 | |  |  |  |  |
| Patient-Reported Experience Measure for Addiction Treatment (PREMAT)^30, 31^ | - the Picker Institute’s principles of patient-centred care were used to guide focus group discussions with people accessing five different AOD treatment programs.(p.665)^30^ - The second aim of this study was to develop a PREM for AOD treatment settings based on the themes of the focus group discussions. This involved constructing questionnaire items that were subsequently reviewed by consumers and service providers of AOD treatment services. (p.665)^30^ - This process involved developing a topic guide for focus groups, conducting focus groups, constructing a draft measure based on focus group analysis and having an expert panel review the measure. (p.665)^30^ - The researchers were aware of the potential for social distance between themselves as researchers (KH, PK) and a past service provider (PK) to impact participant willingness to talk openly about experiences. To address this, they explicitly emphasised the vital importance of understanding and learning from the authentic consumer experience. (p.665)^30^ - Focus groups involved asking participants openended questions and additional probing questions that explored the participants’ experiences of the eight domains in AOD treatment services. (p.665)^30^ - Data coding and analysis were conducted using iterative categorisation, a technique developed for Patient experiences of AOD treatment analysing qualitative data within the addiction field. The Picker Institute domains of patient-centred care were used as deductive codes in the preliminary coding framework. Coded data were reviewed line by line to identify inductive sub-themes that were incorporated into the coding framework. KH developed the initial codes, and then, throughout significant points of the data analysis, KH and PK met to discuss emerging codes and categories and the interpretation of texts.(pp.665-666)^30^ - KH [researcher] translated each subtheme into two positively worded statements rated on a 5-point Likert scale. Two open-ended questions were developed to capture additional experiences. The researchers then collectively reviewed the items to discern if they adequately captured the sub-themes until agreement was reached. This process resulted in a 36-item draft PREM. (p.666)^30^ - consumers and providers of AOD treatment services were then invited to provide feedback on the draft PREM. One of the AOD treatment services within close proximity to the researchers was approached to convene a consumer focus group. Following staff invitation, seven people living with substance use disorders agreed to participate, and KH attended the service to obtain written informed consent and conduct the focus group. Participants completed a demographic and background questionnaire and reviewed the draft PREM. Open-ended questions and additional probing questions invited the participants to refine the questionnaire items by commenting on wording and content and to provide feedback on the response options and the questionnaire layout. Concurrently, providers of the AOD treatment services were emailed an invitation to review the draft PREM attached as a Microsoft Word document. Service providers were purposively selected to cover a different range of settings and therapeutic approaches. They were informed of the aims and methods of the research and asked to provide any feedback they thought was helpful using tracked changes. (p.666)^30^ - The draft PREM was then revised by the researchers based on the collective feedback from consumers and service providers. Decisions on the final item wording and selection were focused around maximising comprehensibility, acceptability and relevance. Any differences in opinion between consumers and service providers were resolved with these considerations in mind. (p.666)^30^ - Overall, participants had positive impressions of the PREM for Addiction Treatment (PREMAT). The draft PREM was revised based on feedback and resulted in a 33-item measure, including 31 statements and two open-ended questions (Table 4). The statements are rated on a Likert scale that has the following anchors: ‘strongly agree’, ‘agree’, ‘neither agree nor disagree’, ‘disagree’ and ‘strongly disagree’. Consumer and service provider participants agreed that the instructions and layout were appropriate for the AOD treatment setting. People accessing AOD treatment. One item was added following participant feedback. (p.667)^30^ - As such, consumers were involved at all stages of questionnaire development, which enhanced the content validity of the measure. (p.672)^30^ - The inclusion of open ended items further enhances the content validity of the measure by allowing the opportunity to capture patient experiences that are not included in the PREMAT statements. The importance of this opportunity is highlighted by the significant discussion from consumers and service providers around the items of involvement of family and friends and access to medications. It is not surprising that these domains attracted debate given that they are inherently complex, multifaceted and emotionally charged issues. As such, it seemed that their measurement is more appropriate on a case-by-case basis when it is personally meaningful to the consumer, and the open-ended items provide the opportunity to collect such information. Overall, it was considered that the review of the PREMAT by consumers and service providers demonstrated promising indications of good face and content validity. (p.672)^30^ - This draft version of the PREMAT was then additionally reviewed by people accessing AOD treatment and providers from a range of settings and therapeutic approaches, and a final 33-item PREMAT was established (p.2)^31^ |  |  |  |  | 🗸 |  | 🗸 |  |  | 🗸 |  |  |  | 🗸 |  | 🗸 | 🗸 |  |  | | 🗸 |  |  |  |
| South African Addiction Treatment Services Assessment (SAATSA), and an adaptation for adolescents^32, 33^ (note: the original measure did not involve people accessing AOD services in its development, but the later adolescent version did) | - First, a national steering committee identified five domains and corresponding indicators on which treatment quality should be assessed. A decision was made to develop a patient survey to assess several of these indicators. A stakeholder work group sourced survey items and generated additional items where appropriate. The feasibility and face validity of these items were examined during cognitive response testing with 16 patients. This led to the elimination of several items. Next, we conducted an initial psychometric validation of the SAATSA with 364 patients from residential and outpatient services. Exploratory (EFA) and confirmatory factor analyses (CFA) were conducted to assess the latent structure of the SAATSA. Findings highlighted areas where the SAATSA required revision. Following revision, we conducted another psychometric validation with an additional sample of 285 patients. We used EFA and CFA to assess construct validity and we assessed reliability using Cronbach’s measure of internal consistency. (p.1)^32^ - A national steering committee, comprising key stakeholders and role players from the substance abuse treatment field, was formed to identify domains on which to assess treatment. This committee reviewed the domains used in other mental health and substance abuse performance measurement systems, and debated the relevance of these domains for South African services. The steering committee identified five domains on which treatment should be assessed: effectiveness, efficiency, access to treatment, person-centred services, and quality of services. Next, the committee generated a set of indicators (30 in total) that could be used to measure each domain. A Delphi consensus panel comprising 36 content experts, was used to reduce the number of indicators. Of these stakeholders, 26 were substance abuse treatment providers, four were researchers and six were service planners. (p.3)^32^ - Each expert was asked to use a three-point scale (1 = low, 3 = high) to rate each indicator on how important each was an indicator of substance abuse treatment outcomes or quality of care, and feasibility of measurement. The research team calculated the mean importance, mean feasibility and a combined score for each indicator (using an importance: feasibility mean score ratio of 1:2) and ranked the indicators in order of their combined scores. The combined scores were used to reduce the number of indicators: those with a rating of 6.4 or higher (on a scale of 3 to 9) were retained, reducing the number of indicators to 18. After reducing the number of indicators, the steering committee decided whether to measure this indicator using a patient survey, data from treatment records or administrative data typically collected by treatment programmes. A description of the domains and corresponding indicators is provided in Table 1. (p.3)^32^ - From the steering committee, we developed a workgroup (comprising the research team and representatives from residential and outpatient treatment facilities) to generate a list of items to include in the patient survey. Based on a review of the literature, the survey workgroup identified the 18-item US Substance Abuse Perceptions of Care Survey as a tool that could be adapted for use in South Africa. Where this survey did not address our indicators, committee members generated additional items. These additional items related to changes in sexual risk behavior, social connectedness, quality of life, and access to services. After generating several new items, the SQM steering committee made decisions about which items would be retained for the next stage of questionnaire development. This reduced the original set of 41 items to 33 items. Next, decisions were made about the instrument’s layout, the wording of instructions and items, and the response format. In this version of the SAATSA, all items were rated on a four point scale, with response options ranging from “strongly disagree” (1) to “strongly agree” (4). Each item also had a “not applicable” response option. Finally, we conducted a lexile analysis and revised the wording of items until every item was at a Grade 8 reading level. (p3)^32^ - Next, the SAATSA was assessed for feasibility and face validity during cognitive response testing. Face validity refers to whether the instrument appears on face value to measure the key constructs it is purported to measure. The SAATSA was administered to 16 patients receiving substance use treatment between March and June 2011. (p.3)^32^ - During cognitive response testing, patients were instructed to respond to each item as if they were completing the survey, and to identify items that were difficult to read or confusing. The length of time taken to complete the SAATSA was recorded. Patients were asked a series of questions about the clarity of the instructions and the items, what they understood they were being asked, suggestions for alternative wording of items, items that should be added or removed, and the overall format of the instrument. (pp.3-4)^32^ - Results of cognitive response testing Most participants reported that the SAATSA was easy to complete (94 %) and understand (94 %). All participants reported that they could complete the SAATSA with very little assistance. Based on participants’ responses to the items, several changes were made to the SAATSA (p.5)^32^ - Through a participatory and consensus-driven process, treatment stakeholders identified a set of domains and corresponding indicators for assessing substance abuse treatment quality. Next we created questionnaire items to measure indicators for these domains. The face validity of the questionnaire was established through review by experts, and through cognitive response testing with service users. The construct validity and initial reliability of the SAATSA was established during two rounds of pilot-testing with service users. (p.11)^32^ - First, we did not include former patients in the national steering committee, primarily because until very recently, substance abuse treatment service users in South Africa were not well organised into consumer interest groups. We acknowledge that treatment service users may have had different views of quality, however we believe we have mitigated this limitation through conducting considerable outreach to patients during the developmental phases of the SAATSA. In these phases, we were able to capture patients’ perceptions and understanding of treatment quality. Since these early developmental phases, the steering committee has been expanded to include former patients. (p.13)^32^ - These PROMs and PREMs were co-developed with adult service users who, as experts in their lived experience, provided inputs into several stages of the development process to ensure that the SAATSA measured outcomes and experiences that service users consider important.(p.824)^33^ - Between April and June 2018, we conducted five focus groups with adolescents (13–17 years old) receiving SUD treatment. (p.825)^33^ - Two master’s-level project staff (one male and one female) with training and experience in conducting qualitative research facilitated the group discussions. A semistructured discussion guide, developed within the SQM Initiative’s national steering committee, was used to facilitate discussions around experiences of treatment, perceptions of treatment quality and desired outcomes. (p.825)^33^ - Adolescent service users identified several additional concepts they thought should be incorporated into the questionnaire to enhance the relevance and meaningfulness of the proposed measures. They also provided recommendations for improving the wording of questions and their administration to enhance their acceptability and the likelihood of their completion. (p.828)^33^ - This feedback is being used to improve the clarity and comprehensibility of items for greater acceptability. (p.829)^33^ |  |  |  |  | 🗸 |  | 🗸 |  |  | 🗸 |  |  |  | 🗸 |  |  |  | 🗸 | 🗸 | |  |  |  |  |
| Un-named measure developed for the ‘Downtown Eastside Second Generation Strategy’ (DTES-2GS)^34^ | - Our community partners included three peer-led organizations of PWUD which are based in Vancouver’s Downtown Eastside (DTES) and have long-standing research partnerships with the BC-CfE and BCCSU. The Vancouver Area Network of Drug Users (VANDU) is the largest peer-based drug user organization in Canada… The Western Aboriginal Harm Reduction Society (WAHRS) is a group of urban Aboriginal PWUD focused on developing support, education and training programs that reflect values of Aboriginal people. The BC Association of People on Methadone (BCAPOM) is a peer-based support and advocacy group for people receiving methadone and other forms of opioid agonist treatment. (p.17)^34^ - The academic research team approached the community groups in October 2016 to share initial plans for an evaluation of the DTES-2GS and to request their involvement as community partners. Members of the board of directors provided support for the project and expressed interest in being actively involved in designing the study. It was imperative to the community partners that they not merely be consulted but be active participants with meaningful influence over the research process. Toward this end, we secured financial support to carry out a workshop series with members of the community partners to identify issues that these organizations would like addressed in the evaluation and refine development of a patient-reported experience questionnaire. (p.17)^34^ - The academic research team undertook an iterative and collaborative process with the community partners to plan the workshop series. An initial list of domains for the questionnaire were identified by the academic research team through a review of existing patient reported experience measures, drawing primarily from the WHO responsiveness survey. These domains were then used to structure an initial set of openended questions to guide dialogue with the community partners about health care experiences and access in the DTES. Workshop agendas were further refined by peers through discussions at a VANDU board meeting and three planning sessions held with board members of WAHRS. (pp.17-18)^34^ - We hosted three workshop sessions at the VANDU office to guide development of the questionnaire. In total, 18 participants were selected through a nomination process coordinated by the director of VANDU and the president of WAHRS. An approximately even number of men and women participated in the workshop series. To be nominated, participants had to be active members of the drug user organizations and be able to communicate in English. The sessions were audio-recorded with the consent of participants, and three members of the academic research team attended as observers. (p.18)^34^ - For the first workshop, a facilitator led nine members from the community groups in a dialogue about patient experience and health service access in the DTES. Members of the academic research team opened the session by providing a brief description of the DTES-2GS, the existing research cohorts, and the rationale for the patient experience questionnaire. The remainder of the session followed a focus group format to elicit input from participants on topics related to health care access and patient experience that should be included on this questionnaire (Figs. 1, 2). Participants were asked to share their opinions on health care services in the DTES and how quality of services could be evaluated from a client perspective. (p.18)^34^ - Indigenous methodologies were used in the development and execution of the second workshop, a talking circle convened with nine Indigenous members of WAHRS. (p.18)^34^ - Following the first two workshop sessions, three members of the academic research team conducted a thematic analysis of the transcriptions and observer notes. From this analysis, key themes within a number of health care domains were identified and the WHO responsiveness questionnaire was adapted to ensure key aspects of participant health care experiences were captured, forming the first draft of the supplemental questionnaire. All participants from the first two workshops were invited to attend a report-back session at VANDU with the purpose of (a) communicating how the research team had incorporated workshop discussions into the preliminary design on the questionnaire; (b) gauging the feasibility and acceptability of the components covered in the questionnaire; (c) gathering direct feedback on the structure, language, and clarity of the draft questionnaire; and (d) receiving suggestions on alternative or additional questions to be included. The session was formatted as a discussion circle led by the study principal investigators. The session was co-facilitated by a local Elder, who provided a blessing for the session and closing remarks. Drafted questions were presented one at a time and displayed on an overhead projector. For each question, a brief introduction was provided of the domain and how it related to key themes from the workshop, followed by a discussion to examine both content and structure. Handouts of the drafted questions were distributed to provide participants, who had the option of providing feedback anonymously. Feedback from this session informed a second wave of revisions to the supplemental questionnaire. (pp.18-19)^34^ - While questionnaire development was led by independent (non- VCH) investigators and community partners, VCH representatives were consulted in the development of the questionnaire to ensure that it adequately addressed key components of the DTES-2GS. Specifically, VCH input was required to ensure that the questionnaire focused on health care services and providers that would be impacted by the DTES-2GS, and that the questionnaire provided VCH with actionable information to inform adaptation and measurement of progress over time. VCH representatives provided direct feedback on the questionnaire content, structure, and language to further refine a subsequent draft. (pp.19-20)^34^ - Feedback from workshop participants on the design of the questionnaire, both at the report back session and during pilot testing, was important to ensure that the instrument was appropriate to the unique needs of PWUD in the DTES. In terms of structure, participants affirmed the inclusion of domains from the WHO responsiveness survey and highlight domains of quality of care that were most impactful on their health and well-being for more in-depth data collection. Participants identified the need to break down more complex questions or add and remove response options based on their experiences of care in the DTES. Participants identified areas where language could be simplified to improve comprehensibility of questions, while also identifying language that should be avoided as it was felt to be discriminatory toward PWUD (e.g., avoiding the term ‘substance use disorder’). Most importantly, participants provided examples based on their experiences to help clarify key concepts in each quality of care domain addressed in the questionnaire. For example, participants provided examples of what it means to be treated with respect by a health care provider (e.g., they make you feel welcome, ask how you are doing, and remember your name). (p.21)^34^ |  | 🗸 |  |  |  | 🗸 | 🗸 |  | 🗸 | 🗸 |  | 🗸 | 🗸 |  |  | 🗸 | 🗸 |  |  | | 🗸 |  |  |  |

**References**

1. Strobbe S, Brower KJ and Galen LW. Patient satisfaction with outpatient detoxification from alcohol. *Journal of Addictions Nursing* 2004; 15: 23-29.

2. Madden A, Lea T, Bath N, et al. Satisfaction guaranteed? What clients on methadone and buprenorphine think about their treatment. *Drug and Alcohol Review* 2008; 27(6): 671-678.

3. Stöver H. Barriers to opioid substitution treatment access, entry and retention: a survey of opioid users, patients in treatment, and treating and non-treating physicians. *European Addiction Research* 2011; 17: 44-54.

4. Fischer G and Stover H. Assessing the current state of opioid-dependence treatment across Europe: Methodology of the European quality audit of opioid treatment (EQUATOR) project. *Heroin Addiction and Related Clinical Problems* 2012; 14(3): 5-70.

5. Eurasian Harm Reduction Association. *Survey of client satisfaction with opioid maintenance therapy (OMT) services among patients of OMT programmes in Kyiv and the Kyiv Oblast region. Pilot study report*. 2020. Vilnius, Lithuania: EHRA.

6. Kirouac M, Witkiewitz K and Donovan DM. Client Evaluation of Treatment for Alcohol Use Disorder in COMBINE. *Journal of Substance Abuse Treatment* 2016; 67: 38-43.

7. Ives M, Funk R, Ihnes P, et al. *GAIN Global Appraisal of Individual Needs Evaluation Manual*. 2012. Illinois: GAIN Coordinating Center, Chestnut Health Systems.

8. Congia P, Tarantini F, Paolo Pani P, et al. Exploring components and correlates of satisfaction in an outpatient drug addiction service. *Italian Journal on Addiction* 2012; 2: 6-12.

9. Best D. *The NTA's 2005 survey of user satisfaction in England*. 2007. London: National Treatment Agencyfor Substance Misuse.

10. Forman R, Crits-Christoph P, Kaynak Ö, et al. A feasibility study of a web-based performance improvement system for substance abuse treatment providers. *Journal of substance abuse treatment* 2007; 33: 363-371.

11. Hogan B, Hershey L and Ritchey S. A case study using a patient satisfaction survey to improve the delivery and effectiveness of drug addiction treatment services: marketing implications and organizational impact. *Health marketing quarterly* 2007; 24(1-2): 93-106.

12. Eisen SV, Wilcox M, Idiculla T, et al. Assessing consumer perceptions of inpatient psychiatric treatment: the perceptions of care survey. *The Joint Commission journal on quality improvement* 2002; 28(9): 510-526.

13. Barry DT, Moore BA, Pantalon MV, et al. Patient satisfaction with primary care office-based buprenorphine/naloxone treatment. *J Gen Intern Med* 2007; 22: 242-245.

14. Kehoe P, Wodak A and Degenhardt L. *Patient satisfaction in a NSW public opioid pharmacotherapy clinic: measurement and responses*. National Drug and Alcohol Research Centre, University of New South Wales, 2004.

15. Petrar S, Kerr T, Tyndall MW, et al. Injection drug users' perceptions regarding use of a medically supervised safer injecting facility. *Addictive Behaviors* 2007; 32(5): 1088-1093.

16. Alcohol Tobacco and Other Drug Association ACT (ATODA). *Service Users' Satisfaction and Outcomes Survey 2018: a census of people accessing specialist alcohol and other drug services in the ACT*. 2020. Canberra: ATODA.

17. Howard J and Arcuri A. Predictors of retention, and client perceptions of treatment satisfaction and outcomes, among young people presenting to residential drug and alcohol treatment with alcohol as a primary or secondary substance of concern. *Australia: Ted Noffs Foundation* 2005.

18. Cole TO, Robinson D, Kelley-Freeman A, et al. Patient Satisfaction With Medications for Opioid Use Disorder Treatment via Telemedicine: Brief Literature Review and Development of a New Assessment. *Frontiers in public health* 2021; 8: 557275.

19. Joe GW, Broome KM, Rowan-Szal GA, et al. Measuring patient attributes and engagement in treatment. *Journal of substance abuse treatment* 2002; 22: 183-196.

20. Marsden J, Stewart D, Gossop M, et al. Assessing client satisfaction with treatment for substance use problems and the development of the treatment perceptions questionnaire (TPQ). *Addict Res Theory* 2000; 8: 455-470. Article.

21. Stallvik M, Flemmen G, Salthammer JA, et al. Assessing health service satisfaction among users with substance use disorders within the municipalities in Norway. *Subst Abuse Treat Prev Policy* 2019; 14: 18.

22. Dhumal T. *Patient Satisfaction with Substance Use Disorder Rehabilitation Services*. M.S., Duquesne University, Ann Arbor, 2019.

23. Lea T, Sheridan J and Winstock A. Consumer satisfaction with opioid treatment services at community pharmacies in Australia. *Pharm World Sci* 2008; 30: 940-946.

24. Perez de los Cobos J, Valero S, Haro G, et al. Development and psychometric properties of the Verona Service Satisfaction Scale for methadone-treated opioid-dependent patients (VSSS-MT). *Drug Alcohol Depend* 2002; 68: 209-214.

25. HSE National Addiction Advisory Governance Group. *National Summary of the Service User Experience Survey 2017*. 2018. HSE National Social Inclusion Office.

26. Rush B, Hansson E, Cvetanova Y, et al. *Development of a client perception of care tool for mental health and addictions: Qualitative, quantitative, and psychometric analysis: Final report for the Ministry of Health and Long-Term Care*. Centre for Addiction and Mental Health, 2014.

27. Liu P, Currie S and Adamyk-Simpson J. What are the most important dimensions of quality for addiction and mental health services from the perspective of its users? *Patient Experience Journal* 2018; 5: 106-114.

28. Currie SR, Liu P, Adamyk-Simpson J, et al. Validation of a Comprehensive Patient Experience Survey for Addiction and Mental Health that was Co-designed with Service Users. *Community Mental Health Journal* 2020; 56: 735-743.

29. Haugum M, Iversen HH, Bjertnaes O, et al. Patient experiences questionnaire for interdisciplinary treatment for substance dependence (PEQ-ITSD): Reliability and validity following a national survey in Norway. *BMC Psychiatry* 2017; 17(1) (no pagination).

30. Hinsley K, Kelly PJ and Davis E. Experiences of patient-centred care in alcohol and other drug treatment settings: A qualitative study to inform design of a patient-reported experience measure. *Drug and alcohol review* 2019; 38(6): 664-673.

31. Kelly PJ, Hatton EL, Hinsley K, et al. Preliminary psychometric evaluation of the patient reported experience measure for addiction treatment (PREMAT). *Addict Behav* 2021; 123: 107048.

32. Myers B, Govender R, Koch JR, et al. Development and psychometric validation of a novel patient survey to assess perceived quality of substance abuse treatment in South Africa. *Substance abuse treatment, prevention, and policy* 2015; 10: 44.

33. Myers B, Johnson K, Lucas W, et al. South African service users' perceptions of patient-reported outcome and experience measures for adolescent substance use treatment: A qualitative study. *Drug Alcohol Rev* 2019; 38: 823-830.

34. Olding M, Hayashi K, Pearce L, et al. Developing a patient-reported experience questionnaire with and for people who use drugs: A community engagement process in Vancouver's Downtown Eastside. *International Journal of Drug Policy* 2018; 59: 16-23.
